# Classification accuracy of structural and functional connectomes across different depressive phenotypes

**DOI:** 10.1162/imag_a_00064

**Published:** 2024-01-17

**Authors:** Hon Wah Yeung, Aleks Stolicyn, Xueyi Shen, Mark J. Adams, Liana Romaniuk, Gladi Thng, Colin R. Buchanan, Elliot M. Tucker-Drob, Mark E. Bastin, Andrew M. McIntosh, Simon R. Cox, Keith M. Smith, Heather C. Whalley

**Affiliations:** Department of Psychiatry, University of Edinburgh, Edinburgh, United Kingdom; Department of Psychology, University of Edinburgh, Edinburgh, United Kingdom; Lothian Birth Cohorts, University of Edinburgh, Edinburgh, United Kingdom; Scottish Imaging Network, A Platform for Scientific Excellence Collaboration (SINAPSE), Edinburgh, United Kingdom; Department of Psychology, University of Texas at Austin, Austin, TX, United States; Population Research Center and Center on Aging and Population Sciences, University of Texas at Austin, Austin, TX, United States; Centre for Clinical Brain Science, University of Edinburgh, Edinburgh, United Kingdom; Centre for Genomic and Experimental Medicine, Institute of Genetics and Molecular Medicine, University of Edinburgh, Edinburgh, United Kingdom; Department of Computer and Information Sciences, University of Strathclyde, Glasgow, United Kingdom

**Keywords:** depression phenotyping, classification modelling, functional connectomes, structural connectomes

## Abstract

Phenotyping of major depressive disorder (MDD) can vary from study to study, which, together with heterogeneity of the disorder, may contribute to the inconsistent associations with neuroimaging features and underlie previous problems with machine-learning methods for MDD diagnostic applications. In this study, we examined the classification accuracy of structural and functional connectomes across different depressive phenotypes, including separating MDD subgroups into those with and without self-reported exposure to childhood trauma (CT) (one of the largest risk factors for MDD associated with brain development). We applied logistic ridge regression to classify control and MDD participants defined by six different MDD definitions in a large community-based sample (N=14,507). We used brain connectomic data based on six structural and two functional network weightings and conducted a comprehensive analysis to (i) explore how well different connectome modalities predict different MDD phenotypes commonly used in research, (ii) whether stratification of MDD based on self-reported exposure to childhood trauma (measured with the childhood trauma questionnaire (CTQ)) may improve the accuracies, and (iii) identify important predictive features across different MDD phenotypes. We found that functional connectomes outperformed structural connectomes as features for MDD classification across phenotypes. The highest accuracy of 64.8% (chance level 50.0%) was achieved in the Currently Depressed (defined by the presence of more than five symptoms of depression in the past 2 weeks) sample with additional CTQ criterion using partial correlation functional connectomes. The predictive feature overlap, measured using Jaccard index, indicated that there were neurobiological differences between MDD patients with and without childhood adversity. Further analysis of predictive features for different MDD phenotypes with hypergeometric tests revealed sensorimotor and visual subnetworks as important predictors of MDD. Our results suggest that differences in sensorimotor and visual subnetworks may serve as potential biomarkers of MDD.

## 1 Introduction

Major depressive disorder (MDD) is a disabling psychiatric condition which affects a substantial proportion of the general population around the world. In the clinical setting, diagnosis of MDD relies on clinical examination of signs and symptoms, and taking a detailed history from the patient and collateral sources. This necessarily involves a strong subjective element. These clinical features are aligned against multiple diagnostic criteria, which in the case of MDD as defined by the Diagnostic and Statistical Manual of Mental Disorders - V (DSM-V) gives rise to more than 200 qualifying symptom combinations (Østergaard et al., 2011; Zimmerman et al., 2015). This is further complicated in the research setting where different assessment tools are often used from one research study to another, particularly in larger studies where full clinical assessments are not possible, and this makes the diagnosis of MDD problematic. The use of different definitions of MDD likely exacerbates inconsistencies in findings and may underlie problems with using imaging data to classify MDD accurately. Recently, the use of mental health questionnaire-based items to efficiently categorise mood disorder allows researchers to thoroughly explore how depression phenotypes defined with different methods are associated with environmental risk factors, genetics, and neuroimaging measures. The large samples now available, together with increased computational capabilities and machine-learning techniques, could be key to improving classification and are a step towards understanding depression heterogeneity and potential subtyping.

In the context of Genome Wide Association analyses, Howard et al. (2018) investigated three definitions of MDD—broad depression, probable MDD, and International Classification of Diseases based MDD—in UK Biobank (UKB) sample. They reported high genetic correlations among the three MDD phenotypes, indicating a potential core genetic component shared across the MDD definitions. They also found genetic associations that were specific to each MDD phenotype (Howard et al., 2018). Cai et al. (2020) presented five definitions of MDD in the same sample; three of the definitions were categorised as minimally phenotyped, and the other two were termed as strictly-defined according to clinical diagnostic criteria (Cai et al., 2020). The authors showed through clustering analysis that the broader definitions exhibited distinct patterns of associations with environmental risk factors as compared to more strictly defined MDD. They also found that the stricter definitions of MDD exhibited higher heritability estimates, more specific genetic architecture, and differing patterns of genetic associations with the strictly defined phenotypes. In terms of analyses of functional brain imaging data, some studies have indicated that associations may be specific to different depressive symptoms. For example, rumination (Kühn et al., 2012; Wu et al., 2015; Zhu et al., 2012), helplessness and hopelessness (Peng et al., 2014; Yao et al., 2009), and suicidal tendencies (Fan et al., 2013; S. Zhang et al., 2016) were found to be associated with abnormal functional connectivity of different brain regions (Brakowski et al., 2017). While in structural brain imaging, MDD-related differences were found to be more consistent across depression phenotypes. In a previous work, Harris et al. (2022) studied the structural brain differences, measures for the whole brain (cortical thickness, cortical volume, and subcortical volume), cortical lobes, and white matter tract types (fractional anisotropy and mean diffusivity), for three main MDD definitions from self-report to clinical definitions in N=39,300 UKB imaging participants (Harris et al., 2022). The study found that the associations with white matter integrity were consistent across depression phenotypes and that phenotype-specific differences were more prominent in cortical thickness measures, despite small overall effect sizes. These studies primarily investigated the influence of phenotyping methods using univariate approaches. In contrast, multivariate analyses and machine learning (ML) approaches could provide additional insight by combining information of different features for diagnostic classification. So far, there has not been a large-scale study investigating the effect of depression phenotyping on the results of ML predictive modelling based on structural and functional connectivity data. We hypothesised that our investigated ML models would deploy different decision strategies (reflected in model coefficients), and identify different sets of important features for prediction of different depression phenotypes.

In addition to examining the effects of varying depression phenotyping methods, we here also examine the effect of stratifying depression by presence or absence of early life adversity. Previous studies have shown that early life adversity is associated with increased risk of developing psychiatric disorders, including depression in adulthood (Kuzminskaite et al., 2021; Mandelli et al., 2015). Levels of childhood adversity have also been associated with subsequent severity of depression and anxiety symptoms (Huh et al., 2017) as well as abnormal brain connectivity in MDD (Grant et al., 2014; Yu et al., 2019). Luo, Chen, Li, Wu, Lin, Yao, Yu, Wu, et al. (2022) investigated functional connectivity differences between healthy controls (N=80) and MDD cases with and without childhood trauma (N=31 and N=30, respectively). Although both MDD groups had similar alterations in functional connectivity compared to controls, the changes appeared more prominent in cases with self-reported childhood trauma. Specifically, MDD cases with self-reported childhood trauma exhibited a larger decrease in connectivity between the ventral attention network and sensorimotor network (Luo, Chen, Li, Wu, Lin, Yao, Yu, Wu, et al., 2022). These results suggest that MDD cases with self-reported childhood trauma may represent a more homogeneous subgroup of MDD, which could be more amenable to diagnostic classification using ML algorithms.

In this study, we investigated diagnostic classification of MDD using connectivity data from the UKB and logistic ridge regression model. We tested the effects of MDD phenotyping on model performance and on the estimated model coefficients. We investigated prediction of six different definitions of MDD using two functional connectome measures (partial correlation and full correlation matrices) and six structural connectome measures. For each diagnostic definition, we maintained a 1-to-1 ratio between cases and controls, with case and control participants matched for age, sex, and intracranial volume (ICV), to enable objective comparison of classification accuracies across models. To investigate whether higher classification accuracies may be achieved for MDD subgroups defined by self-reported childhood trauma, we repeated the above analyses with only selected MDD participants passing the abbreviated CTQ score threshold. Our main aims were (i) to compare ML classification performances between the different MDD definitions and different brain connectivity modalities, (ii) to investigate the effect of childhood trauma score thresholding on classification accuracies, (iii) to identify the important features for classifying each MDD phenotype, based on model coefficients, that could potentially inform biologically/mechanistically distinct subgroups, and (iv) to define important brain subnetworks that may be useful for classifying MDD in general (i.e., important subnetwork changes that are common to different MDD phenotypes).

## 2 Materials and Methods

### 2.1 Materials

Participants were recruited and brain imaging was completed as part of the UKB study. The six depression phenotypes were derived based on mental health questionnaire (MHQ) items. Five MHQ items were used to derive the abbreviated CTQ score.

#### 2.1.1 Participants

A subset of the UKB participants underwent brain MRI at the UKB imaging centre in Cheadle, Manchester, UK and in Newcastle, UK. The study was approved by the National Health Service Research Ethics Service (No. 11/NW/0382) and by the UKB Access Committee (Project No. 4844 and No. 10279). Written consent was obtained from all participants.

#### 2.1.2 Structural networks

At the time of processing, N=9,858 participants with compatible T1-weighted and dMRI data were available from the UKB, and the structural connectomes for these participants were derived locally. A full description of structural connectome processing can be found in Buchanan et al. (2020) and Yeung et al. (2022). These processes are described briefly below.

All imaging data were acquired using a single Siemens Skyra 3 T scanner (Siemens Medical Solutions, Erlangen, Germany; see http://biobank.ctsu.ox.ac.uk/crystal/refer.cgi?id=2367). Details of the MRI protocol and preprocessing are freely available (Alfaro-Almagro et al., 2018; Miller et al., 2016). Each T1-weighted volume was parcellated into 85 distinct neuroanatomical Regions-Of-Interest (ROI) with FreeSurfer v5.3.0 and 34 cortical structures per hemisphere were identified according to the Desikan-Killany atlas (Desikan et al., 2006). Brain stem, accumbens area, amygdala, caudate nucleus, hippocampus, pallidum, putamen, thalamus, and ventral diencephalon were also extracted with FreeSurfer. Whole-brain tractography was performed using an established probabilistic algorithm (BEDPOSTX/ProbtrackX; (Behrens et al., 2007, 2003)) using criteria as described previously (Buchanan et al., 2020). Water diffusion parameters were estimated for FA, which measures the degree of anisotropic water molecule diffusion, and for MD, which measures the magnitude of diffusion. Neurite orientation dispersion and density imaging (NODDI) provides more detailed characterisation of tissue microstructure, and the measures derived from NODDI were: ICVF which measures neurite density; ISOVF which measures extracellular water diffusion; and OD which measures the degree of fanning or angular variation in neurite orientation (H. Zhang et al., 2012). After aligning ROIs from T1-weighted to diffusion space, networks were then constructed by identifying pairwise connections between the 85 ROIs and represented in the form of person-specific 85×85 adjacency matrices. Six network weightings were computed. Streamline count (SC) was computed by recording the total streamline count (uncorrected) between each pair of ROIs. In addition, five further network weightings (FA, MD, ICVF, ISOVF, and OD) were computed by recording the mean value of the diffusion parameter in voxels identified along all interconnecting streamlines between each pair of ROIs.

In total, 8,183 participants (45.1–78.5 years of age, 3,869 male) remained after participants were excluded following local quality checking or due to failure in processing. Proportional-thresholding was used to keep only connections present in at least 2/3 of subjects (Buchanan et al., 2020; de Reus & van den Heuvel, 2013). 6,247 out of 8,183 participants have completed the MHQ questionnaire.

#### 2.1.3 Resting-state functional networks

N=19,831 participants underwent a resting-state functional MRI (rs-fMRI) assessment and passed the quality check by the UKB. Out of 19,831 participants, 14,507 had completed the MHQ questionnaire.

The functional connectome matrices were derived by the UKB imaging project team. The detailed methods of the imaging processing for UKB can be found in previous protocol articles (Alfaro-Almagro et al., 2018; Miller et al., 2016). The processes are described briefly below.

From raw rs-fMRI scans to the correlation and partial correlation matrices, the data went through steps of data preprocessing, parcellation using group independent component analysis (ICA), and connectivity estimation with FSL packages (http://biobank.ctsu.ox.ac.uk/crystal/refer.cgi?id=1977) by the UKB imaging team. The preprocessing steps were completed in the following sequence: motion correction, grand mean intensity normalisation, high-pass temporal filtering, echo-planar image unwarping, gradient distortion correction unwarping, and finally removal of structured artifacts (Miller et al., 2016). A group level ICA was carried out on the first 4,100 participants and the spatial ICA mask was applied to the fMRI scans, parcellating the brain into 100 components. 45 of the 100 components were identified as noise components and were removed. The time-series data for remaining nodes was then used to calculate functional connectivity between node pairs. The full correlation matrices were computed using normalised temporal correlation of the time-series between each pair of nodes. As for the partial correlation matrices, these were computed using partial Pearson correlation with an L2 regularisation applied (rho set as 0.5 for Ridge Regression in FSLNets). All r-scores were then Fisher-transformed into z-scores. This resulted in two 55 x 55 correlation matrices (correlation and partial correlation) of functional connectivity for each participant. The list of good nodes can be found in: http://www.fmrib.ox.ac.uk/datasets/ukbiobank/group_means/edge_list_d100.txt. An interactive website displaying group-mean maps for each component can be found in: http://www.fmrib.ox.ac.uk/datasets/ukbiobank/group_means/rfMRI_ICA_d100_good_nodes.html. A connectome map of the nodes can be found in: http://www.fmrib.ox.ac.uk/datasets/ukbiobank/netjs_d100/.

##### 2.1.3.1 Mapping functional subnetworks

The 55 brain regions (network nodes) are clustered into six groups according to group mean full correlation matrices. We referred these clusters of ICA nodes as functional subnetworks, and the clusters were consistent with previous studies (Buckner et al., 2009; Dosenbach et al., 2007; Ji et al., 2019; Reineberg et al., 2015; Uddin et al., 2019; Yeo et al., 2011). The clusters approximately represent the sensorimotor (SMN; orange), visual network (VN; blue), executive control and attention network (EC_AN; green), cingulo-opercular network (CON; purple), default mode network (DMN; red), and extended DMN (eDMN; brown).

**Fig. 1.**
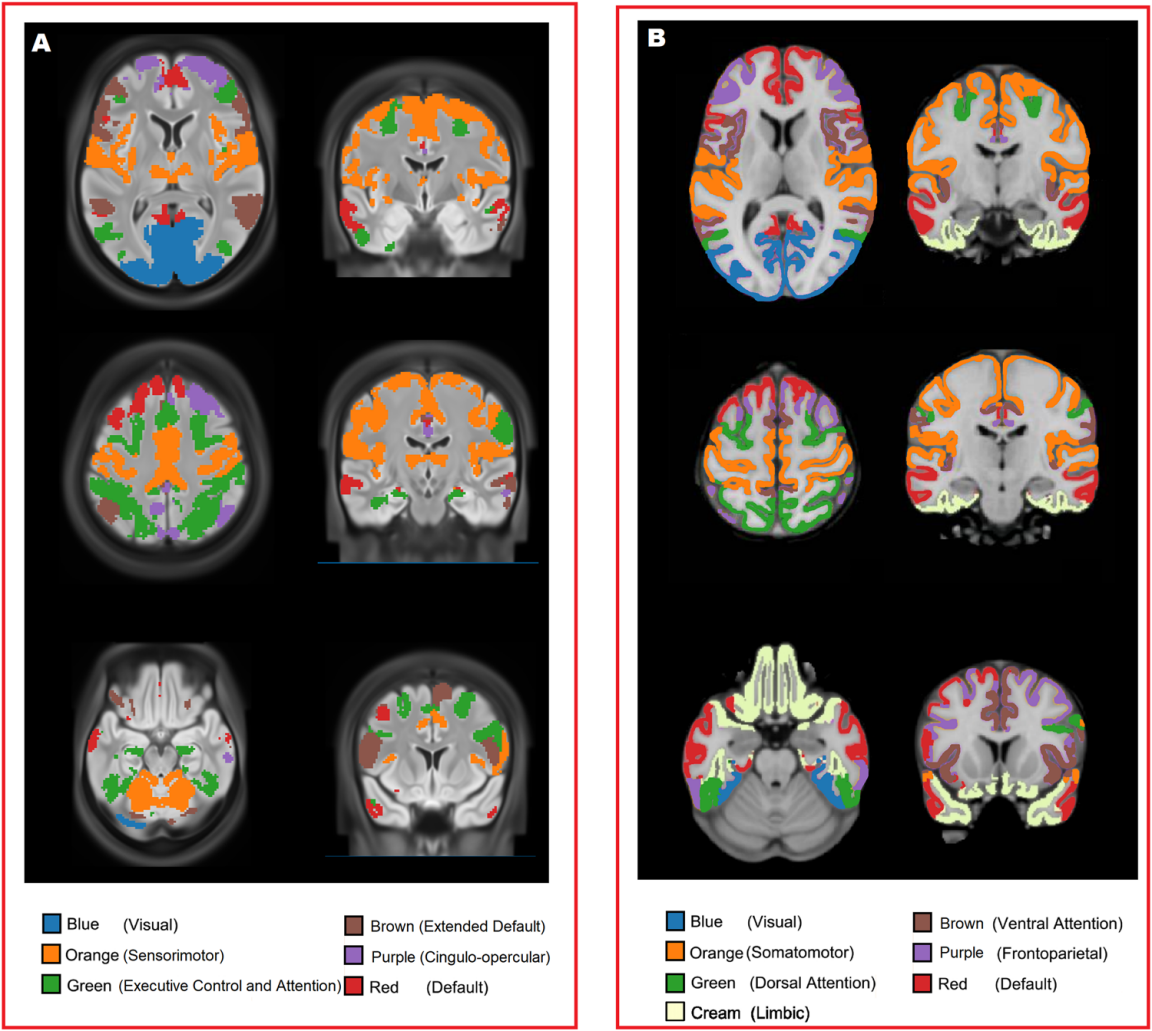
(A) The six functional subnetworks identified in the current study. (B) the reference 7-network parcellation (Yeo et al., 2011), figure adapted from Yeo et al. (2011). The VN, SMN, and DMN roughly map to the corresponding functional subnetworks in the 7-network parcellation. The EC_AN roughly maps to the dorsal attention network as well as parts of the ventral attention network and the FPN in the 7-network parcellation. The CON and eDMN approximately maps to the ventral attention network and the FPN in the 7-network parcellation.

Figure 1A shows the six functional subnetworks, and Figure 1B shows the reference 7-network parcellation proposed by Yeo et al. (2011). The VN, SMN, and DMN roughly map to the corresponding functional subnetworks in the 7-network parcellation. The EC_AN roughly maps to the dorsal attention network as well as parts of the ventral attention network and the FPN in the 7-network parcellation. The CON and eDMN approximately map to the ventral attention network and the FPN in the 7-network parcellation.

#### 2.1.4 Depression phenotypes

A total of 157,357 participants from the UKB completed an online Mental Health Questionnaire (MHQ) including self-report, clinical lifetime disorder status, and experiences of psychiatric symptoms for specific disorders. Davis et al. (2020) presented a detailed implementation for constructing the depression phenotypes from the mental health questionnaire (MHQ) items for UKB (Davis et al., 2020). A number of them have comparatively loose criteria (which may imply mildly depressed) compared with the others. Some of the depression definitions have a large portion of participant overlap. Here, the overlap refers to participants satisfying case criteria for more than one MDD phenotype. We decided to choose one representative (Ever depressed depression phenotype) for the mildly depressed definitions. For the moderate-to-severe MDD phenotypes with a large portion of participant overlap, we chose the more severe definitions (Depression Medicated, Ever Severely Depressed, Currently Depressed, and Recurrent Depression). Participants who have taken antidepressants in the past were defined as cases for the Depression Medicated phenotype. Ever Depressed was based on Composite International Diagnostic Interview (CIDI) diagnostic criteria, where those who reported having at least five depressive symptoms with at least one core symptoms in the past (i.e., having a CIDI severity score of at least 5) were defined as cases for this definition. Cases of Ever Severely Depressed had a CIDI severity score of 8 with current symptoms absent. Currently Depressed cases were those who satisfied the criteria for Ever Depressed and also reported current symptoms. Cases of Recurrent Depression had experienced more than one depressive episodes. We also included another MDD definition, Probable Moderate/Severe Depression, described in Smith et al. (2013). Participants who had experienced at least one two core symptoms for at least 2 weeks and have seen a general practitioner / psychiatrist were defined as cases for Probable Moderate/Severe Depression. In total, we investigated six different depression phenotypes and the detailed descriptions of criteria are in Supplementary Material S1.1, Table S1.

#### 2.1.5 Childhood adversity

The abbreviated CTQ score is derived from a subset of five childhood trauma-related questionnaire items, which is the same set of items used in Davis et al. (2020). The item description and scoring system are provided in Supplementary Material S1.2. Based on research indicating that an aggregated trauma score may provide a better marker of risk for adverse outcomes than individual items (Hughes et al., 2017), we took the average score from the five CTQ items as the final abbreviated CTQ score, which was similar to Warrier and Baron-Cohen (2021). We then investigated the effect of abbreviated CTQ score thresholding on model performance and model coefficients.

Four different abbreviated CTQ score thresholds (None, 0.2, 0.4, and 0.6) corresponding to the 50th, 70th, and 80th quantile, were applied solely on the MDD samples (i.e., MDD cases falling below the CTQ threshold were excluded from the subgroup). The use of different thresholds can help verify the relationship between CTQ severity cut-off and classification accuracies. Table 1 shows the sample size of MDD cases for each definition and for each abbreviated CTQ score threshold.

**Table 1.** The summary characteristic of MDD cases for each MDD phenotype and for each abbreviated CTQ score threshold in UK Biobank. (a) The summary characteristic of MDD cases with functional connectomes available for each MDD phenotype and for each abbreviated CTQ score threshold in UK Biobank.

	No threshold					With CT score ≥0.2					With CT score ≥0.4					With CT score ≥0.6				
Phenotypes	# Cases	/	Sex (F:M)	/	Mean age (in years)	# Cases	/	Sex (F:M)	/	Mean age (in years)	# Cases	/	Sex (F:M)	/	Mean age (in years)	# Cases	/	Sex (F:M)	/	Mean age (in years)
Drug	703	/	514:189	/	62.16	480	/	345:135	/	62.06	382	/	282:100	/	61.95	288	/	209:79	/	61.95
Ever	3323	/	2262:1061	/	61.52	2327	/	1584:743	/	61.33	1718	/	1189:529	/	61.23	1215	/	856:359	/	61.08
Ever Severe	529	/	393:136	/	59.58	427	/	316:111	/	59.72	341	/	259:82	/	59.88	254	/	198:56	/	59.73
Current	200	/	128:72	/	58.67	164	/	103:61	/	58.34	142	/	88:54	/	58.53	112	/	69:43	/	58.43
Recurrent	1926	/	1359:567	/	60.97	1408	/	987:421	/	60.86	1092	/	777:315	/	60.87	788	/	568:220	/	60.71
MDD narrow	902	/	637:265	/	60.07	679	/	470:209	/	60.00	515	/	362:153	/	60.10	368	/	259:109	/	59.72

**Table:** (b) The summary characteristic of MDD cases with structural connectomes available for each MDD phenotype and for each abbreviated CTQ score threshold in UK Biobank.

	No threshold					With CT score ≥0.2					With CT score ≥0.4					With CT score ≥0.6				
Phenotypes	# Cases	/	Sex (F:M)	/	Mean age (in years)	# Cases	/	Sex (F:M)	/	Mean age (in years)	# Cases	/	Sex (F:M)	/	Mean age (in years)	# Cases	/	Sex (F:M)	/	Mean Age (in years)
Drug	313	/	224:89	/	60.88	226	/	156:70	/	60.60	175	/	129:46	/	60.54	137	/	100:37	/	60.27
Ever	1480	/	998:482	/	60.87	1038	/	700:338	/	60.64	777	/	537:240	/	60.55	548	/	391:157	/	60.50
Ever Severe	246	/	191:55	/	59.01	193	/	149:44	/	59.15	153	/	124:29	/	59.17	124	/	105:19	/	58.90
Current	92	/	62:30	/	57.88	78	/	52:26	/	57.45	67	/	44:23	/	57.65	57	/	38:19	/	57.89
Recurrent	828	/	582:246	/	60.28	622	/	435:187	/	60.19	486	/	350:136	/	60.32	352	/	256:96	/	60.19
MDD narrow	435	/	312:123	/	59.38	331	/	232:99	/	59.17	252	/	183:69	/	59.62	187	/	138:49	/	58.90

Drug = Depression Medicated, Ever = Ever Depressed, Ever Severe = Ever Severely Depressed, Current = Currently Depressed, Recurrent = Recurrent Depression without Bipolar Disorder, MDD Narrow = Probable Moderate/Severe Depression.

**Fig. 2.**
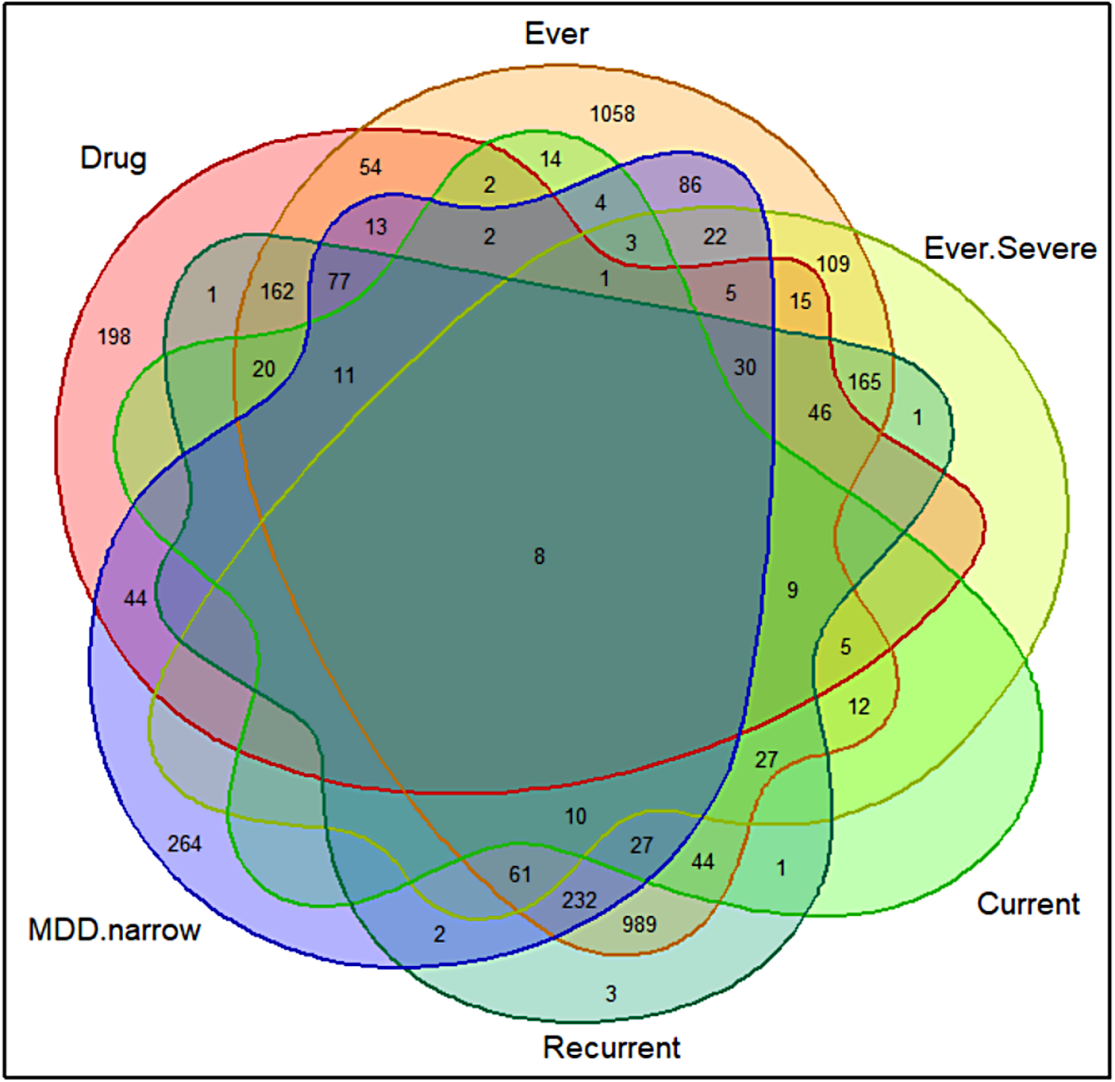
Venn diagram visualisation for the intersections between each MDD phenotypes. Drug = Depression Medicated, Ever = Ever Depressed, Ever.Severe = Ever Severely Depressed, Current = Currently Depressed, Recurrent = Recurrent Depression without Bipolar Disorder, MDD.narrow = Probable Moderate/Severe Depression.

Although the six different MDD phenotyping methods were conceptually different, they were not distinct from each other. The Ever Depressed, Ever Severely Depressed, Currently Depressed, and Recurrent Depression phenotypes were defined using CIDI criteria. The Ever Depressed represented the milder depression type and the other three phenotypes were almost subsets of this milder definition. Moreover, in 50% to 70% of the cases, most of the other phenotypes were defined as cases for the Recurrent Depression phenotype. Table 2 shows the pairwise overlaps among the phenotypes. The diagonal entries show the number of cases that are unique to the phenotype. Details of the intersections between MDD phenotypes are shown in Figure 2.

**Table 2.** The number of pairwise overlapping cases (with percentage of overlap showing in bracket) among the six different MDD phenotypes.

	Drug		Ever		Ever severe		Current		Recurrent		MDD narrow	
Drug	198	(28.2%)	460	(13.8%)	119	(22.5%)	58	(29.0%)	364	(18.9%)	191	(21.2%)
Ever	460	(65.4%)	1058	(31.8%)	528	(99.8%)	199	(99.5%)	1918	(99.6%)	592	(65.6%)
Ever Severe	119	(16.9%)	528	(15.9%)	0	(0.0%)	75	(37.5%)	357	(18.5%)	140	(15.5%)
Current	58	(8.3%)	199	(6.0%)	75	(14.2%)	0	(0.0%)	157	(8.2%)	66	(7.3%)
Recurrent	364	(51.8%)	1918	(57.7%)	357	(67.5%)	157	(78.5%)	3	(0.2%)	458	(50.8%)
MDD narrow	191	(27.2%)	592	(17.8%)	140	(26.5%)	66	(33.0%)	458	(23.8%)	264	(29.3%)
Total	703	(100%)	3323	(100%)	529	(100%)	200	(100%)	1926	(100%)	902	(100%)

The diagonal entries show the number of cases that are unique to the phenotype. Drug = Depression Medicated, Ever = Ever Depressed, Ever Severe = Ever Severely Depressed, Current = Currently Depressed, Recurrent = Recurrent Depression without Bipolar Disorder, MDD Narrow = Probable Moderate/Severe Depression.

### 2.2 Methods

#### 2.2.1 Classification model

Previous neuroimaging ML studies indicate that classical ML models achieved accuracy comparable to Deep Learning (DL) models (He et al., 2020). Moreover, work by Schulz et al., (2020) indicate that simple linear models were just as competitive as non-linear models, sometimes even outperforming non-linear models, in predicting common phenotypes from brain scans (Schulz et al., 2020). We have found similar results in a previous study of structural connectomes that ridge regression tends to be more consistent than other models in terms of model coefficients (Yeung et al., 2022). Therefore, a logistic ridge regression model was chosen for classification modelling in the current study.

#### 2.2.2 Feature inputs

In this study, the inputs to the classification model are the vectorised upper triangular non-zero entries of the connectivity matrices (Number of features: functional connectivity, N=1,485; structural connectivity, N=2,210). We additionally examined combined inputs of functional and structural connectivity to the models, where one of the six structural modalities is stacked on one of the two functional modalities to give a long vector of connectivity features (Number of features, N=3,695). The combinations give 12 sets of combined connectivity features.

#### 2.2.3 Regularisation parameter optimisation

In order to find the optimal regularisation parameter, λ, we tested different values ranging from $1\times 10^{-3}$ to $1\times 10^{3}$. The λ that optimised validation accuracies at each iteration of the inner loop was chosen, so the λs are different for each fold, each MDD definition, and each connectivity modality.

#### 2.2.4 Case-control matching and correction for confounders

We aimed to run classification models with a 1-to-1 ratio between cases and controls for each of the six MDD phenotypes. Those who are not defined as cases in any of the 17 MDD definitions (derived from Davis et al. (2020) and Smith et al. (2013)) formed the full healthy control sample (Full-HC). For each case participant from the six MDD-phenotype samples, we selected a control from the Full-HC matched by sex, with the smallest difference in age and in ICV. In the case with CTQ thresholding, the CTQ criterion was applied to the MDD samples first before case-control matching so that the classification models always run on a sample with a 1-to-1 ratio between MDD cases and healthy controls.

Following the above stringent criteria (particularly with minimal differences in ICV), we confirmed that there was exactly one matched control for each case, and therefore none of the control samples were drawn at random. The study focused on MDD and therefore, participants with other major neurological or psychiatric disorders (namely schizophrenia, bipolar, dissociative identity disorder, autism, intellectual disability, Parkinson’s disease, multiple sclerosis, or cognitive impairment) were excluded.

#### 2.2.5 Experimental setup

We ran a nested cross-validation (CV) to evaluate the performance. In the nested 5-fold CV, the training data (5/6 folds) of each of the six iterations of the outer 6-fold cross-validation were split into 5 folds. Connectome features were z-normalised based on training data. The optimal model, which achieved the highest validation accuracy, was chosen in each of the inner iterations. Therefore, this amounts to a total of 30 evaluations of classification accuracies. The split was carefully done so that the cases and control maintained a 1-to-1 ratio with age, sex, and ICV matched for any of the training, validation, and test set. Test accuracies were averaged across the folds to get performance estimates.

#### 2.2.6 Identifying important features

Model coefficients tell us about feature importance in classification tasks and help us identify the features associated with the specific MDD definition of interest. A simple way to compare feature importance is to observe the magnitudes of the beta coefficients. However, beta coefficients were data-specific and the ranking by beta coefficients may not be generalisable to new data.

In order to identify the features that were truly important, we believed that there were two necessary conditions:

1. The feature should be consistently in the top 50%, by coefficient magnitudes, in all of the 30 models trained in the nested CV.
2. The feature should always have the same coefficient sign in all of the 30 models trained in the nested CV.

#### 2.2.7 Identifying important subnetworks

The majority of biological findings in MDD are of small effects. Evaluation based on aggregated coefficient magnitudes extracted from MDD predictive models is therefore fraught with statistical uncertainty. Therefore, in this study, we compared the number of important features (as defined above) within a subnetwork (or between two subnetworks) to the number expected by random chance. The use of a hypothesis testing offers a more objective measure to assess the importance of a functional subnetwork than a simple aggregation of model coefficients. To check whether there are significantly more/less important features being distributed to certain subnetworks in the connectomes is equivalent to testing hypothesis about probability of success/failure. In most situations, the binomial test is a suitable candidate. However, selecting a feature subset involves sampling without replacement and the probabilities of success among the subnetworks are no longer independent. It is therefore more appropriate to use the hypergeometric test for hypothesis testing. Let N be the number of nodes, $n_{i}$ be the size of subnetwork i, $N_{e}=N\left(N-1\right)/2$ be the number of possible edges in the connectome, K be the total number of important features identified for an MDD phenotype, O be the matrix representing the number of important features between/within subnetworks, and S be the matrix representing the number of possible edges between/within subnetworks is given by:

$$S_{ij}=(\begin{array}{ll} n_{i}\times n_{j}\; & \text{if }i\neq j,\\ n_{i}\left(n_{i}-1\right)/2\; & \text{if }i=j. \end{array}$$

and the p-value matrix, HyperGeom, from the hypergeometric test is given by,


$$HyperGeom_{ij}=(\begin{array}{ll} \sum_{k=0}^{O_{ij}}\frac{\left(\begin{array}{l} S_{ij}\\ k \end{array}\right)\left(\begin{array}{l} N_{e}-S_{ij}\\ K-k \end{array}\right)}{\left(\begin{array}{l} N_{e}\\ K \end{array}\right)} & \text{if }O_{ij}\leq K\frac{S_{ij}}{N_{e}},\\ \sum_{k=O_{ij}}^{K}\frac{\left(\begin{array}{l} S_{ij}\\ k \end{array}\right)\left(\begin{array}{l} N_{e}-S_{ij}\\ K-k \end{array}\right)}{\left(\begin{array}{l} N_{e}\\ K \end{array}\right)} & \text{if}O_{ij}>K\frac{S_{ij}}{N_{e}}. \end{array}$$

## 3 Results

### 3.1 Depression classification performances based on different connectome modalities without CTQ threshold

**Fig. 3.**
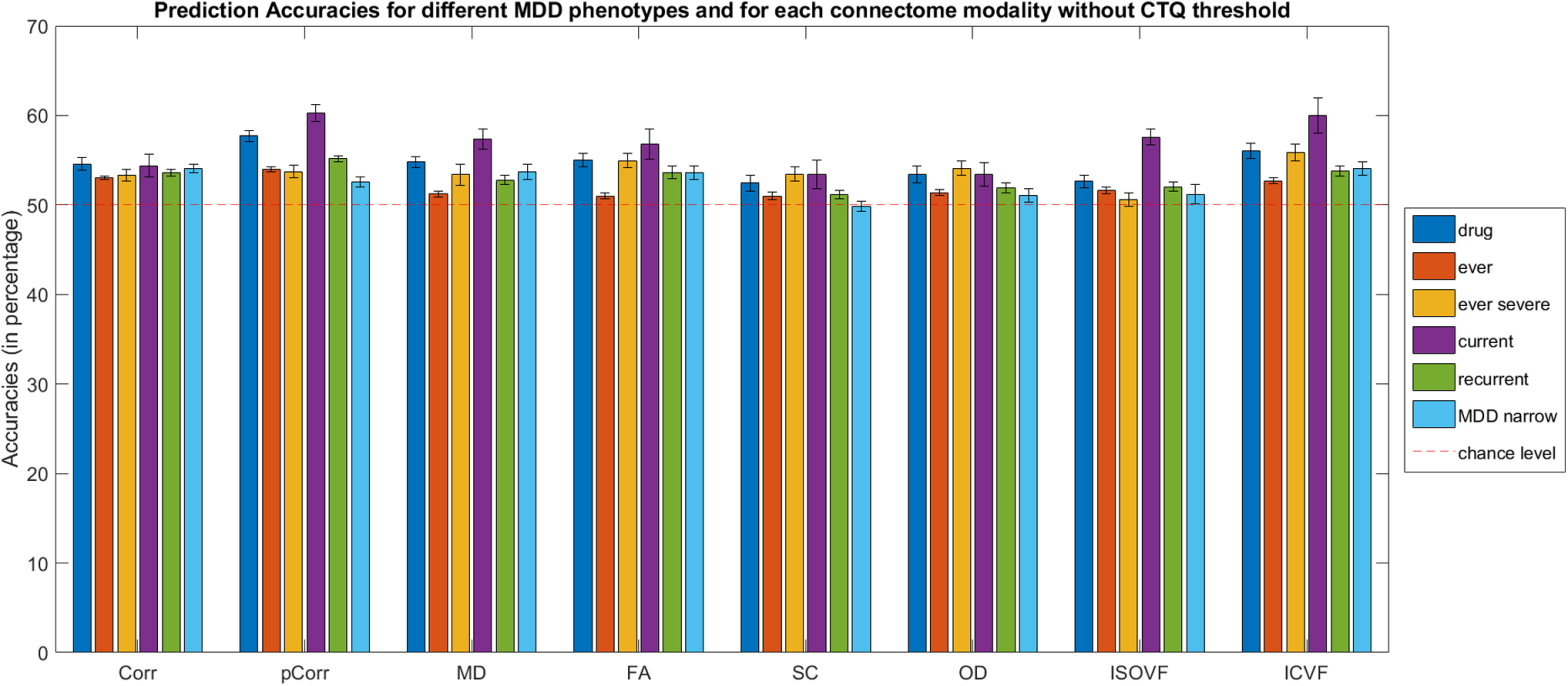
Bar plot for MDD phenotypes (without Childhood Trauma Questionnaire (CTQ) threshold scores) classification accuracies (mean percentage with error bars showing the standard error) with functional connectomes for the test sets. Drug = Depression Medicated, Ever = Ever Depressed, Ever Severe = Ever Severely Depressed, Current = Currently Depressed, Recurrent = Recurrent Depression without Bipolar Disorder, MDD Narrow = Probable Moderate/Severe Depression, Corr = functional correlation connectivity, pCorr = functional partial correlation connectivity, MD = mean diffusivity, FA = fractional anisotropy, SC = streamline count, OD = orientation dispersion, ISOVF = isotropic volume fraction, ICVF = intra-cellular volume fraction.

We applied logistic ridge regression based on structural and functional imaging features to classify the six MDD phenotypes identified above. Figure 3 shows the classification results for different MDD phenotypes based on different connectome modalities and the exact numbers are shown in Supplementary Materials S2, Table S2(a) - (h). The chance-level accuracy of all the classifiers below is 50%.

In terms of functional connectomes, for correlation functional connectivity (Corr), the model classifications for all six MDD definitions had comparable performances, with test accuracies ranging from 53.1% to 54.6%. As for partial correlation functional connectivity (pCorr), the mean test accuracies for Depression Medicated (57.7%) and Currently Depressed (60.2%) outperformed the other definitions. The test accuracies for the other four definitions ranged from 52.6% to 55.2%.

For structural connectomes, the classification accuracies for the test sets (overall range: 49.9–60.0%) were mostly lower than those based on functional connectomes. ICVF gave slightly better overall MDD classification test accuracies (≥52.7%) than the other five network weights (≥49.9%). Similar to findings with functional connectomes, the classification performance for Currently Depressed phenotype was the best among all other phenotypes for all network weights (56.8–60.0%), with the exception of SC and OD (53.4%).

### 3.2 Depression classification performances for currently depressed phenotype based on different connectome modalities with CTQ threshold

**Fig. 4.**
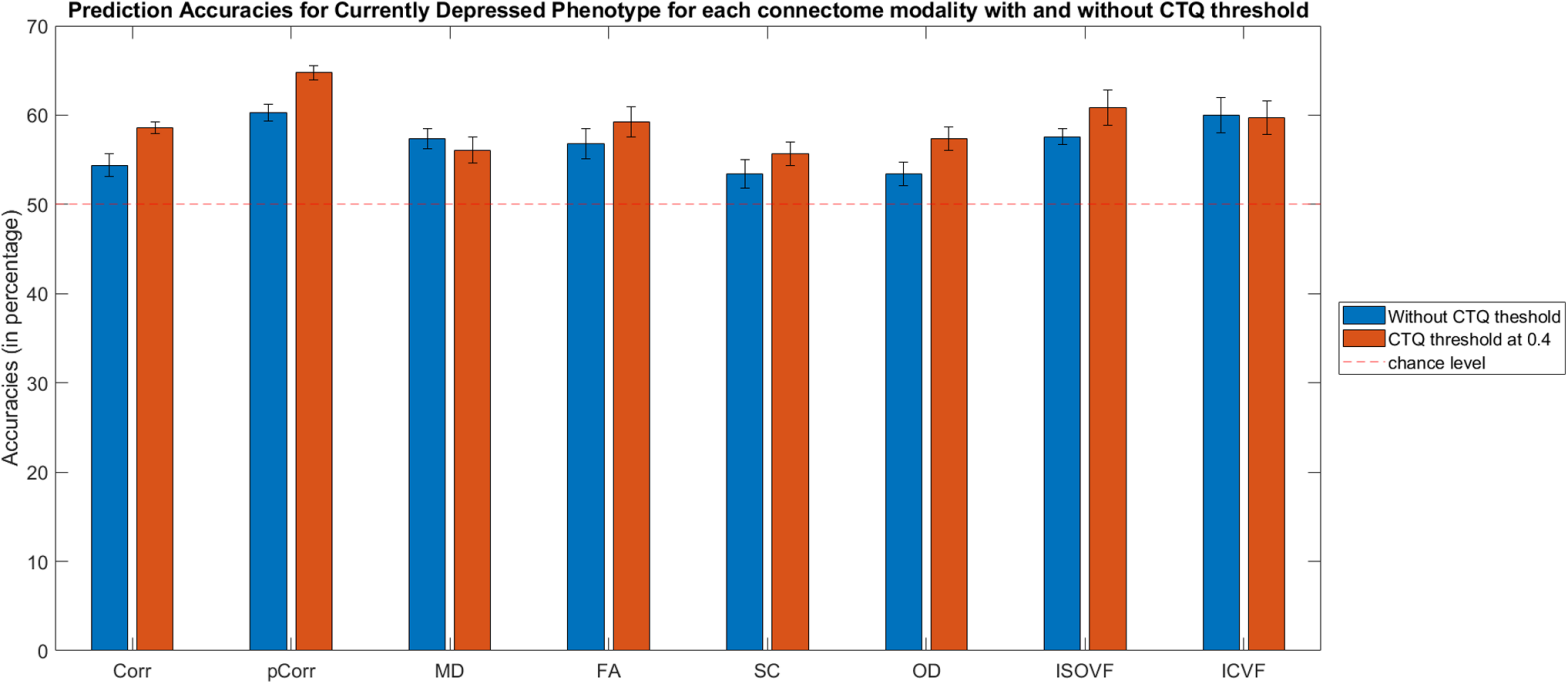
Bar plot for currently depressed phenotype (with and without Childhood Trauma Questionnaire (CTQ) threshold scores) classification accuracies (mean percentage with error bars showing the standard error) with different connectome modalities for the test sets. Corr = functional correlation connectivity, pCorr = functional partial correlation connectivity, MD = mean diffusivity, FA = fractional anisotropy, SC = streamline count, OD = orientation dispersion, ISOVF = isotropic volume fraction, ICVF = intra-cellular volume fraction.

It was found that different connectome modalities generally achieved best test accuracies for the Currently Depressed phenotype, and improvements in classifications were mostly found in models with CTQ threshold at 0.4, see Supplementary Materials S2, Table S2(a) - (h). Figure 4 shows the test accuracies for Currently Depressed phenotype based on different connectome modalities with and without CTQ threshold.

With CTQ threshold, the pCorr gave the best test accuracy (64.8%), with an improvement of 4.6% from the model without CTQ threshold. The MDD classification test accuracies based on Corr improved from 54.4% to 58.6%. The model based on OD and ISOVF also saw significant improvement, with test accuracy rising from 53.4% to 57.4% and from 57.6% to 60.9% respectively. Higher accuracies were also seen in other modalities except for MD and ICVF.

Furthermore, the more severe phenotype (where there were also fewer number of cases) usually exhibited higher accuracies. By checking over all the depression phenotypes and at different levels of CTQ threshold, we observed negative correlations between test accuracies and sample size, and the correlations were statistically significant (i.e., p-value < 0.05) for most of the structural and functional connectivity modalities except for SC, OD, and ISOVF (Corr: r=−0.5152; pCorr: r=−0.4491; MD: r=−0.4140; FA: r=−0.5145; SC: r=−0.2244; OD: r=−0.4021; ISOVF: r=−0.2554; ICVF: r=−0.5514).

Moreover, we note that the female-to-male ratio was approximately 2:1 in all the MDD definitions, see Table 1. We sought to verify if the classification modelling of MDD could benefit from sex stratified models. Since the model performance based on functional connectomes was generally better than those based on other modalities, we additionally built sex-specific MDD classification models based on functional connectomes. The same experimental setup was used for male and female MDD classification models. Details of the test accuracies are in Supplementary Materials S2.1, Table S3. It was found that the original models performed better than the sex-specific ones.

### 3.3 Depression classification performances for currently depressed phenotype based on combined connectivity

We further investigated the added value of structural connectivity to functional connectivity in classification modelling of MDD. Figure 5 shows the classification performances for Currently Depressed phenotypes based on the 12 sets of combined connectivity at different CTQ thresholds. The samples here were restricted to the participants with both functional and structural connectivity data available, and therefore were different from the samples used in the above section. Results showed that adding structural connectivity to Corr boosted the test accuracies (improved by 0.9 - 5.9%, averaged across CTQ thresholds), and the improvements were more prominent for ISOVF (+ 5.8%) and ICVF (+ 5.9%). On the other hand, the added value of structural connectivity to pCorr was not clear. Improvement was only seen with FA and ICVF. Due to the differences in nature of the connectivity (i.e., dynamic vs static), functional and structural connectomes were investigated separately in the following.

**Figure:**
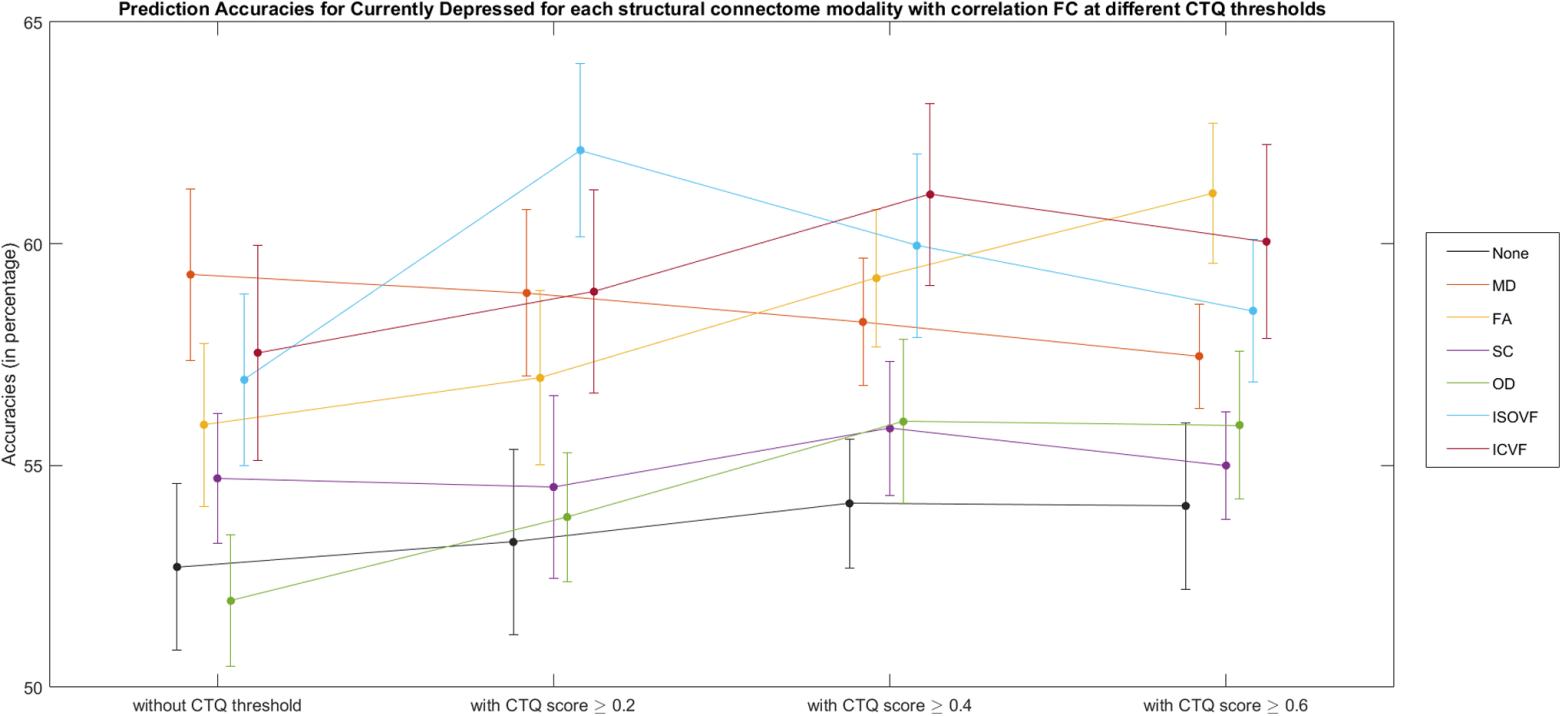
(a) Currently depressed phenotype (at different Childhood Trauma Questionnaire (CTQ) threshold scores) classification accuracies (mean percentage with error bars showing the standard error) based on the combination of correlation functional connectome and each structural connectivity weight for the test sets.

**Figure:**
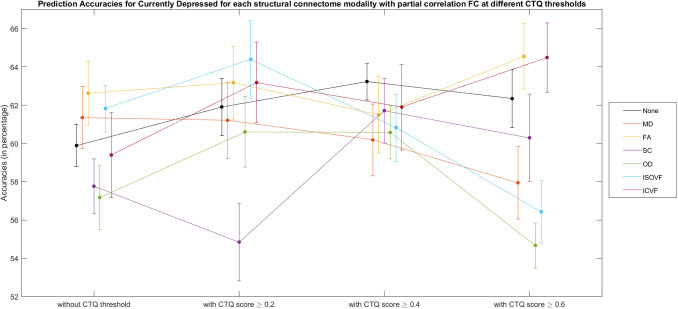
(b) Currently depressed phenotype (at different Childhood Trauma Questionnaire (CTQ) threshold scores) classification accuracies (mean percentage with error bars showing the standard error) based on the combination of partial correlation functional connectome and each structural connectivity weight for the test sets.

**Fig. 5.** Currently depressed phenotype (at different Childhood Trauma Questionnaire (CTQ) threshold scores) classification accuracies (mean percentage with error bars showing the standard error) based on the combination of functional connectome and each structural connectivity weight for the test sets. FC = functional connectome, None = no structural connectivity added to the model, MD = mean diffusivity, FA = fractional anisotropy, SC = streamline count, OD = orientation dispersion, ISOVF = isotropic volume fraction, ICVF = intra-cellular volume fraction. Note that the samples here are restricted to the participants with both functional and structural connectivity data available.

### 3.4 Comparing important edges with and without CTQ threshold

As mentioned in the Methods, we used the two criteria (i.e., feature coefficients consistently with top 50% in magnitude ranking and with same sign across the CV models) to identify important features for the specific MDD phenotype for each imaging modality. In general, we found that the number of important features identified was positively and significantly correlated with models’ test accuracies for most of the functional and structural connectome weights, except for ICVF (Corr: r=0.6553; pCorr: r=0.6544; MD: r=0.4599; FA: r=0.6932; SC: r=0.6118; OD: r=0.5685; ISOVF: r=0.4786; ICVF: r=0.2647), and was negatively and significantly correlated with the sample size for most of the functional and structural connectome weights, except for pCorr (Corr: r=−0.6617; pCorr: r=−0.0630; MD: r=−0.5830; FA: r=−0.8149; SC: r=−0.8398; OD: r=−0.8021; ISOVF: r=−0.6563; ICVF: r=−0.5931). Since the CTQ threshold at 0.4 generally performed the best out of the three thresholds, we chose to compare the important features from models without CTQ threshold and models with CTQ threshold at 0.4.

Table 3 shows the number of important edges identified for each of the depression phenotypes and for each connectome modality as well as the number of feature overlaps between models with and without the CTQ threshold. We also presented the Jaccard index which provided a more objective estimate of the degree of overlap that accounts the differences in the number of important features identified by the models. Formulation of the Jaccard index is presented in the Supplementary Materials S1.3. We found that all models have less than 10% of the edges being identified as robust and important features for predicting depression phenotypes. Moreover, the set of important features identified in the models without CTQ threshold were mostly different from those identified in the models with CTQ threshold (Jaccard =0.07−0.38). A more detailed comparison of the important functional connectome features for the different MDD phenotypes (with and without CTQ threshold) is shown in the following subsection.

**Table 3.** Number of important features identified by the models for different depression phenotypes (with and without CTQ threshold at 0.4) based on different connectome modalities.

MDD phenotype	Drug	Ever	Ever severe	Current	Recurrent	MDD narrow
# Important Feature (without CTQ threshold)	36	10	32	60	16	36
# Important Feature (with CTQ threshold 0.4)	38	29	30	63	22	58
Number of Overlap	10	7	11	23	4	13
Jaccard index	0.1563	0.2188	0.2157	0.2300	0.1176	0.1605

(a) CORRELATION FUNCTIONAL CONNECTOME						
MDD phenotype	Drug	Ever	Ever severe	Current	Recurrent	MDD narrow
# Important Feature (without CTQ threshold)	73	88	48	76	99	48
# Important Feature (with CTQ threshold 0.4)	68	27	50	79	34	55
Number of Overlap	23	13	22	35	18	13
Jaccard index	0.1949	0.1275	0.2895	0.2917	0.1565	0.1444

(b) PARTIAL CORRELATIONS FUNCTIONAL CONNECTOMES						
MDD phenotype	Drug	Ever	Ever severe	Current	Recurrent	MDD narrow
# Important Feature (without CTQ threshold)	89	51	62	89	51	68
# Important Feature (with CTQ threshold 0.4)	97	58	80	95	79	112
Number of Overlap	34	13	27	51	19	18
Jaccard index	0.2237	0.1354	0.2348	0.3835	0.1712	0.1111

(c) MEAN DIFFUSIVITY STRUCTURAL CONNECTOME						
MDD phenotype	Drug	Ever	Ever severe	Current	Recurrent	MDD narrow
# Important Feature (without CTQ threshold)	107	37	90	96	60	88
# Important Feature (with CTQ threshold 0.4)	73	68	72	121	91	90
Number of Overlap	28	9	28	54	21	25
Jaccard index	0.1842	0.0938	0.2090	0.3313	0.1615	0.1634

(d) FRACTIONAL ANISOTROPY STRUCTURAL CONNECTOME						
MDD phenotype	Drug	Ever	Ever severe	Current	Recurrent	MDD narrow
# Important Feature (without CTQ threshold)	80	25	86	96	38	59
# Important Feature (with CTQ threshold 0.4)	74	41	88	100	63	57
Number of Overlap	29	5	29	52	10	14
Jaccard index	0.2320	0.0820	0.2000	0.3611	0.1099	0.1373

(e) STREAMLINE COUNT STRUCTURAL CONNECTOME						
MDD phenotype	Drug	Ever	Ever severe	Current	Recurrent	MDD narrow
# Important Feature (without CTQ threshold)	96	51	85	125	60	75
# Important Feature (with CTQ threshold 0.4)	97	60	84	112	75	102
Number of Overlap	33	16	33	50	24	22
Jaccard index	0.2063	0.1684	0.2426	0.2674	0.2162	0.1419

(f) ORIENTATION DISPERSION STRUCTURAL CONNECTOME						
MDD phenotype	Drug	Ever	Ever severe	Current	Recurrent	MDD narrow
# Important Feature (without CTQ threshold)	79	71	69	95	57	74
# Important Feature (with CTQ threshold 0.4)	84	72	78	105	66	104
Number of Overlap	27	16	28	44	17	28
Jaccard index	0.1985	0.1260	0.2353	0.2821	0.1604	0.1867

(g) ISOTROPIC VOLUME FRACTION STRUCTURAL CONNECTOME						
MDD phenotype	Drug	Ever	Ever severe	Current	Recurrent	MDD narrow
# Important Feature (without CTQ threshold)	83	58	90	74	60	103
# Important Feature (with CTQ threshold 0.4)	88	31	92	116	107	63
Number of Overlap	30	6	38	47	30	25
Jaccard index	0.2128	0.0723	0.2639	0.3287	0.2190	0.1773
(h) INTRA-CELLULAR VOLUME FRACTION STRUCTURAL CONNECTOME						

Drug = Depression Medicated, Ever = Ever Depressed, Ever Severe = Ever Severely Depressed, Current = Currently Depressed, Recurrent = Recurrent Depression without Bipolar Disorder, MDD Narrow = Probable Moderate/Severe Depression.

### 3.5 Significant feature occurrence in subnetworks for different MDD phenotypes

Given that better classification accuracies were achieved with functional connectomes, the following analysis focuses on functional connectomes. Figures 6 and 7 show the results from the hypergeometric test and the normalised aggregated predictive coefficients for the subnetwork functional connectivity. The p-values and normalised predictive coefficients. The are also presented in Supplementary Materials S3, Table S4 - S7. Benjamini-Hochberg procedure was used to correct for FDR (Benjamini & Hochberg, 1995). Details and formulation of the normalised predictive coefficients are presented in Supplementary Materials S1.4.

**Figure:**
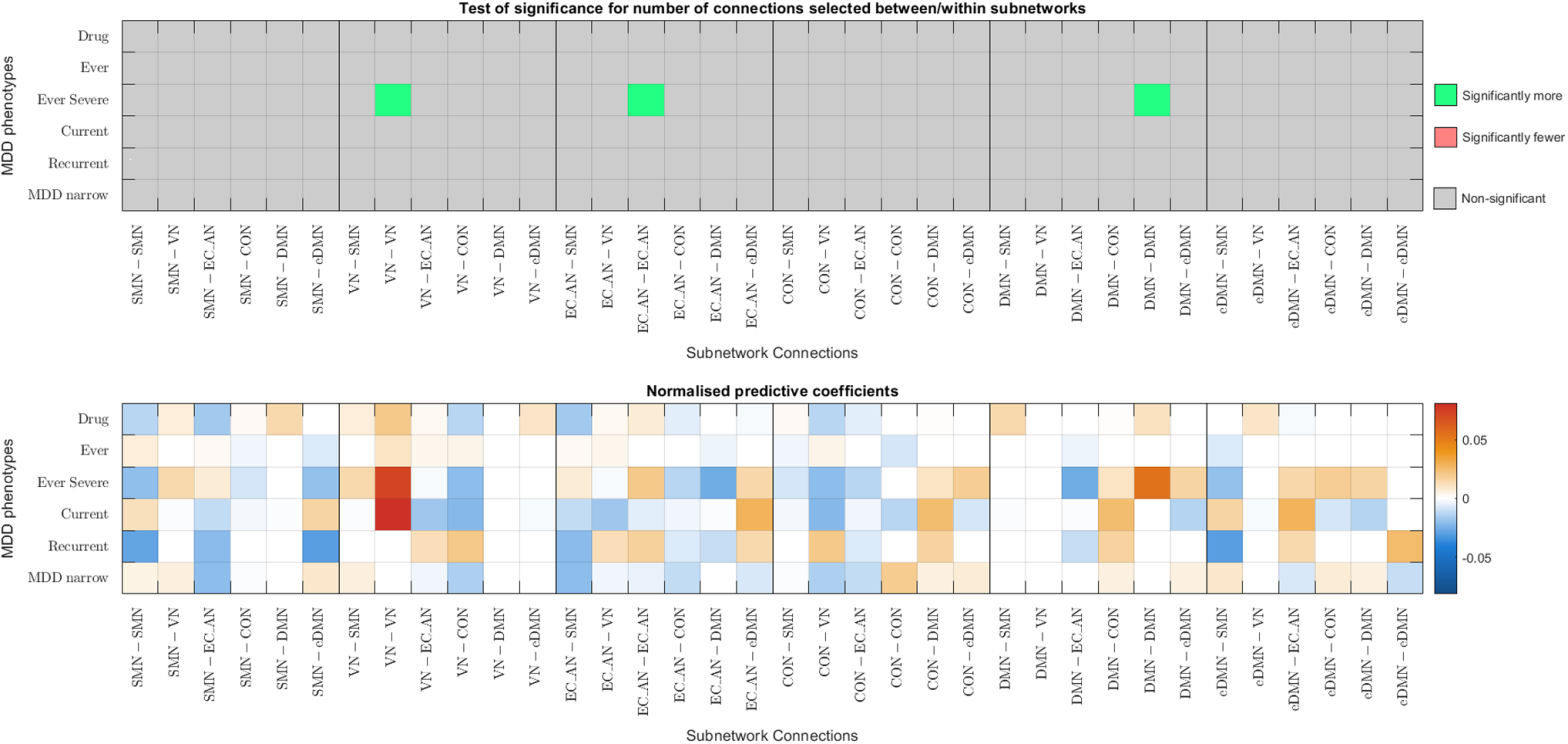
(a) Significance testing of feature occurrence and normalised predictive coefficients between or within subnetworks for the six MDD classification without CTQ threshold.

**Figure:**
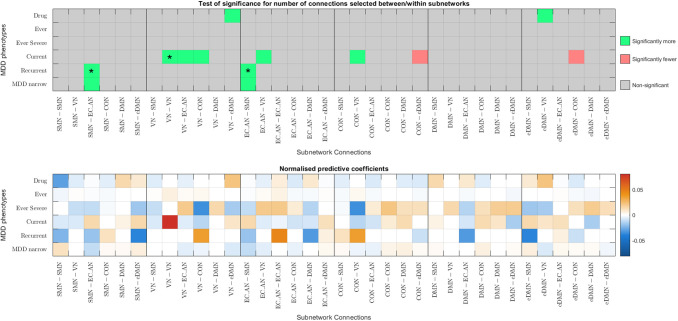
(b) Significance testing of feature occurrence and normalised predictive coefficients between or within subnetworks for the six MDD classification with CTQ threshold at 0.4

**Fig. 6.** Significance testing of feature occurrence and normalised predictive coefficients between or within subnetworks for the six MDD classification with and without CTQ threshold based on Corr functional connectomes. The uncorrected p-values < 0.05 were highlighted in red (for significantly fewer edges) or green (for significantly more edges). *p-values that survived the FDR corrected threshold. Drug = Depression Medicated, Ever = Ever Depressed, Ever Severe = Ever Severely Depressed, Current = Currently Depressed, Recurrent = Recurrent Depression without Bipolar Disorder, MDD Narrow = Probable Moderate/Severe Depression.

**Figure:**
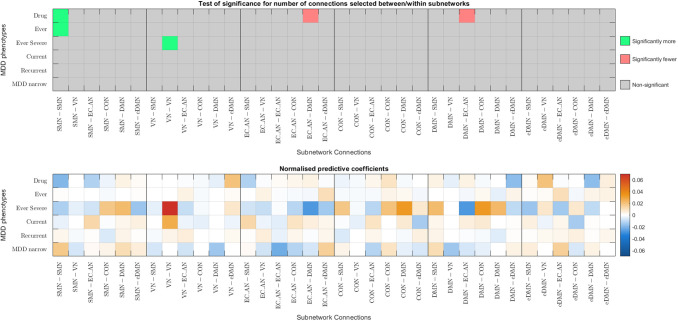
(a) Significance testing of feature occurrence and normalised predictive coefficients between or within subnetworks for the six MDD classification without CTQ threshold.

**Figure:**
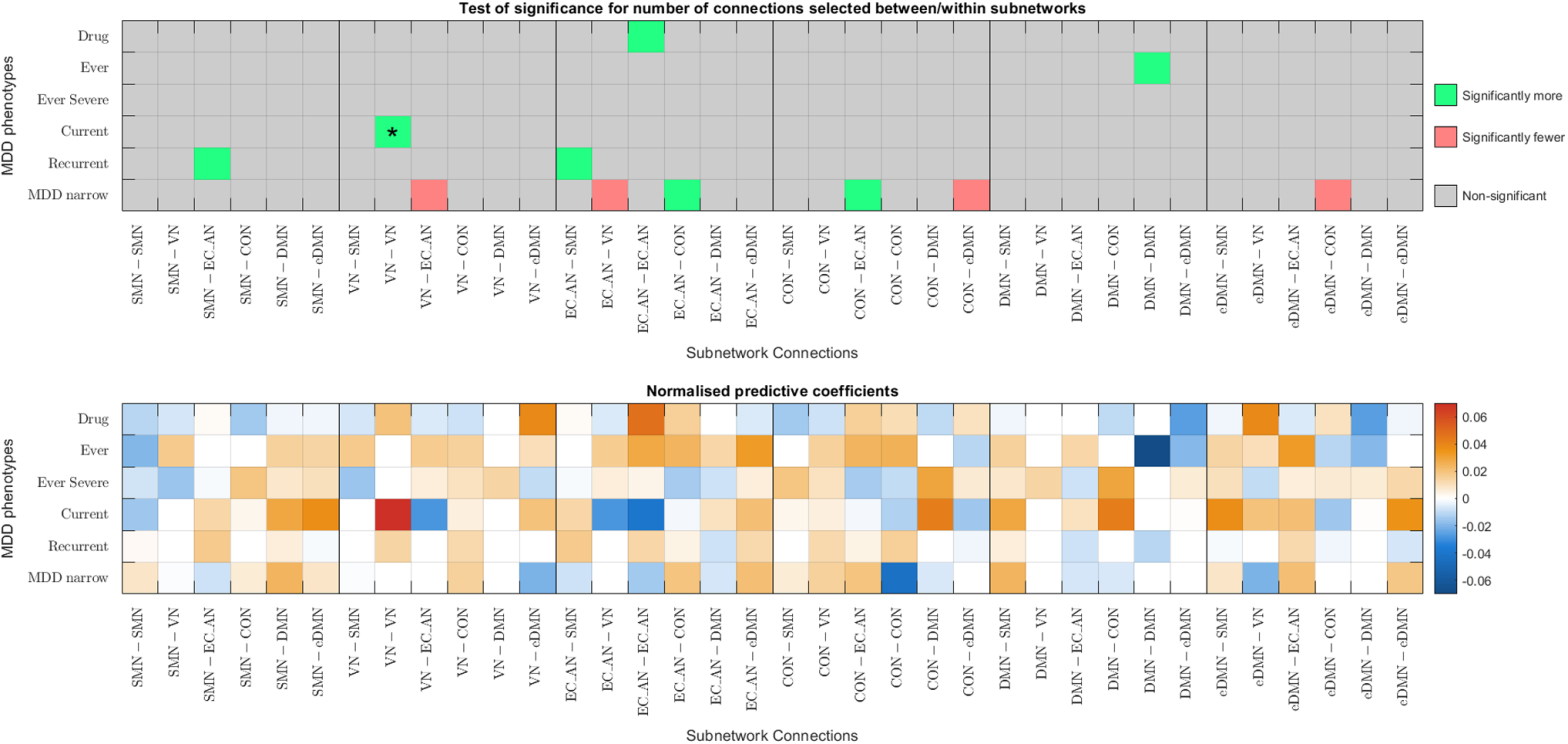
(b) Significance testing of feature occurrence and normalised predictive coefficients between or within subnetworks for the six MDD classification with CTQ threshold at 0.4

**Fig. 7.** Significance testing of feature occurrence and normalised predictive coefficients between or within subnetworks for the six MDD classification with and without CTQ threshold based on pCorr functional connectomes. The uncorrected p-values < 0.05 were highlighted in red (for significantly fewer edges) or green (for significantly more edges). *p-values that survived the FDR corrected threshold. Drug = Depression Medicated, Ever = Ever Depressed, Ever Severe = Ever Severely Depressed, Current = Currently Depressed, Recurrent = Recurrent Depression without Bipolar Disorder, MDD Narrow = Probable Moderate/Severe Depression.

#### 3.5.1 Correlation functional connectomes

Without CTQ threshold, all the p-values were larger than the FDR-corrected threshold. According to the uncorrected p-values, more edges were selected as important features from the connections within VN, within EC_AN, and within DMN were positively predictive for Ever Severely Depressed phenotype (p=0.0182−0.0397, $\beta_{norm}=0.0204-0.0725$), see Figure 6a.

With CTQ threshold, the hypergeometric test showed that there were significantly more edges selected as important features from the connections within VN and positively predictive for Currently Depressed phenotype ($p=0.0005,\beta_{norm}=0.0874$). Significantly more edges were selected as important features from the connections between SMN and EC_AN, and were negatively predictive for Recurrent Depression phenotype (p=0.0062,$\beta_{norm}=-\;\;0.0189$). The rest did not survive the FDR-corrected threshold. According to the uncorrected p-values, more edges were selected as important features from between VN and eDMN and were positively predictive for Depression Medicated phenotype ($p=0.0256,\;\;\;\;\beta_{norm}=0.0228$). More edges were selected as important features from between VN and EC_AN as well as between VN and CON, and were negatively predictive for Currently Depressed phenotype ($p=0.0386-0.0484,\;\beta_{norm}=\;\;-$0.0131−-  0.0061). More edges were selected as important features from between SMN and EC_AN and were negatively predictive for Probable Moderate/Severe Depression phenotype ($p=0.0067,\beta_{norm}=\;$- 0.0111). Fewer edges were selected from connections between CON and eDMN (p=0.0270), see Figure 6b.

#### 3.5.2 Partial correlation functional connectomes

Without CTQ threshold, all the p-values were larger than the FDR-corrected threshold. According to the uncorrected p-values, more edges were selected as important features from the connections within SMN for two MDD phenotypes (p=0.0203−0.0459). They were negatively predictive for Depression Medicated phenotype ($\beta_{norm}=\;$- 0.0194), but were positively predictive for Ever Depressed phenotype ($\beta_{norm}=0.0011$). More edges were selected as important connections from within VN and were positively predictive for Ever Severely Depressed phenotype ($p=0.0390,\beta_{norm}=0.0701$). Fewer edges were selected from between EC_AN and DMN for Depression Medicated phenotype (p=0.0424), see Figure 7a.

With CTQ threshold, the hypergeometric test showed that there were significantly more edges selected as important features from the connections within VN and positively predictive for Currently Depressed phenotype ($p=0.0012,\beta_{norm}=0.0741$). The rest did not survive the FDR-corrected threshold. According to the uncorrected p-values, more edges were selected as important features from within EC_AN for Depression Medicated phenotype (p=0.0421), between SMN and EC_AN for Recurrent Depression phenotype (p=0.0239), and between EC_AN and CON for Probable Moderate/Severe Depression phenotype (p=0.0343). They were positively predictive for MDD (β=0.0175−0.0474). More edges were selected as important features and were negatively predictive for Ever Depressed phenotype ($p=0.0289,\beta_{norm}=\;$-  0.0706). Fewer edges were selected from between VN and EC_AN and between CON and eDMN for Probable Moderate/Severe Depression phenotype (p=0.0431−0.0449), see Figure 7b.

### 3.8 Comparing feature occurrence in subnetworks between models with and without the CTQ threshold for currently depressed phenotype

#### 3.8.1 Correlation matrices

The results showed that significantly more edges were being selected from connections within VN, and between VN and CON for the model with CTQ threshold (CTQmodel). This was similar to the results from the model without the CTQ threshold (NoCTQmodel), where there were more edges being selected from connections, within VN, and between VN and CON (although it did not reach statistical significance for connections between VN and CON). The signs of the normalised aggregated predictive coefficients of these two connections of the NoCTQmodel matched with that of the CTQmodel. More frequently selected edges, although not reaching statistical significance, were selected from between SMN and eDMN, between EC_AN and CON, between CON and DMN, and between DMN and eDMN for both CTQmodel and NoCTQmodel with matching signs.

As for the differences, results showed that the connections between the VN and EC_AN were significantly more frequently selected as important features for the CTQmodel while the same connections were less frequently selected for the NoCTQmodel. The connections between the EC_AN and eDMN were significantly less frequently selected as important features for the CTQmodel while the same connections were more frequently selected for the NoCTQmodel. The connections within CON and within EC_AN were more frequently selected, although not reaching statistical significance, as important features for the NoCTQmodel while the same connections were less frequently selected for the CTQmodel, see Figure 8a – 8b.

**Correlation Functional Connectomes:**
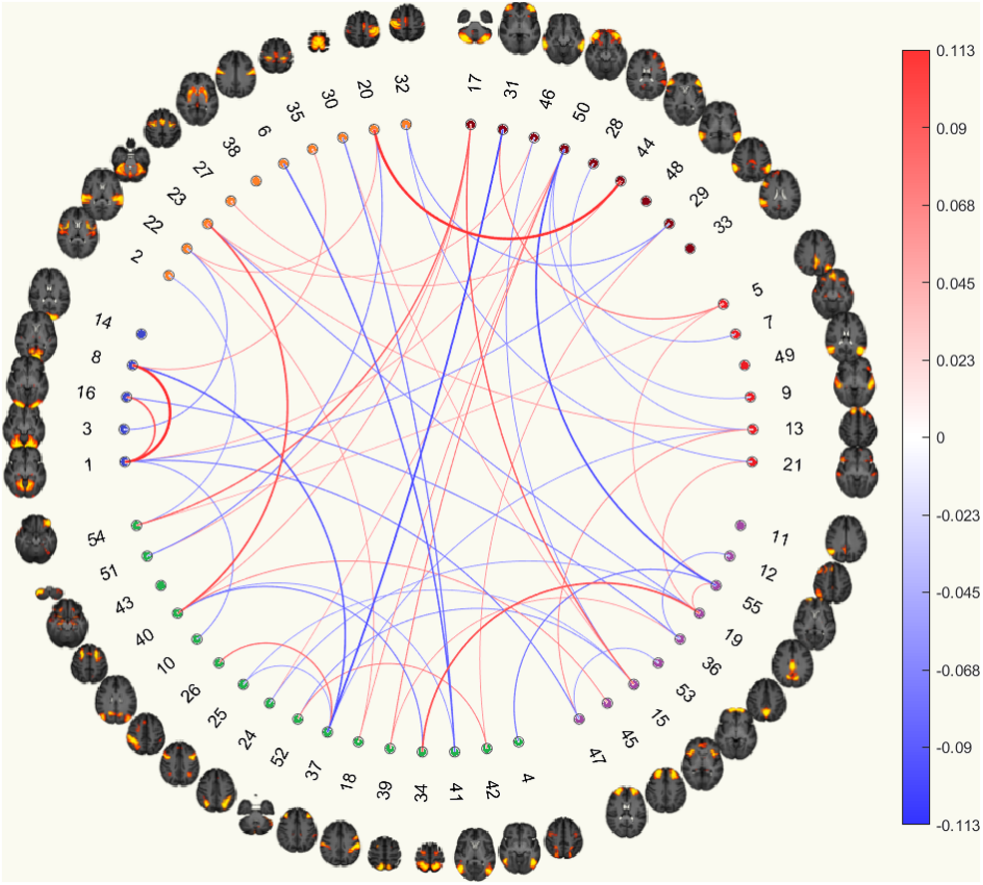
(a) Currently depressed based on correlation functional connectomes.

**Figure:**
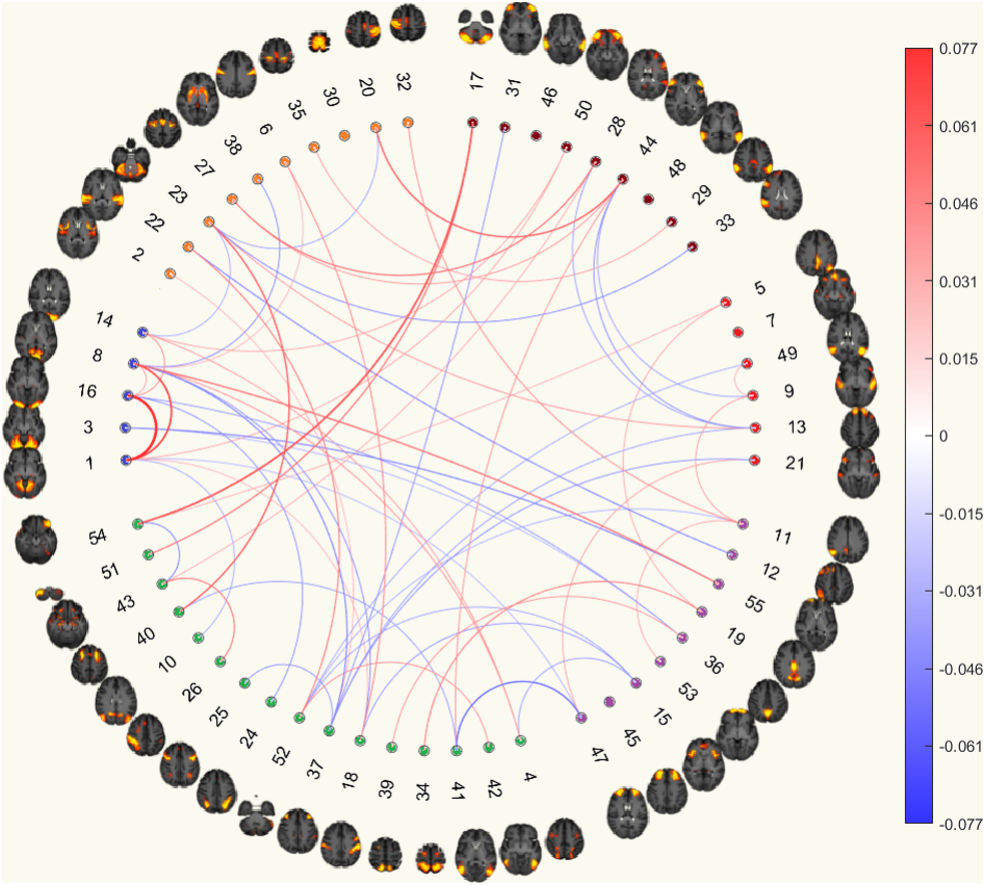
(b) Currently depressed with CTQ threshold based on correlation functional connectomes.

**Partial Correlation Functional Connectomes:**
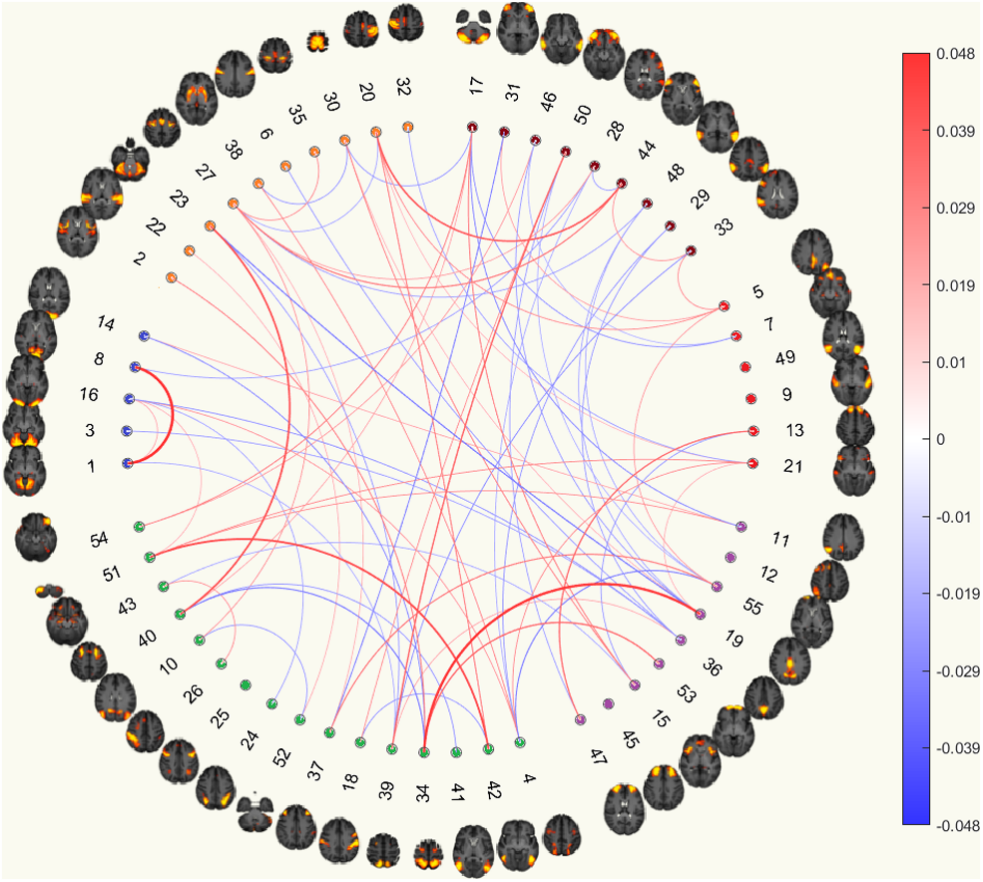
(c) Currently depressed based on partial correlation functional connectomes.

**Figure:**
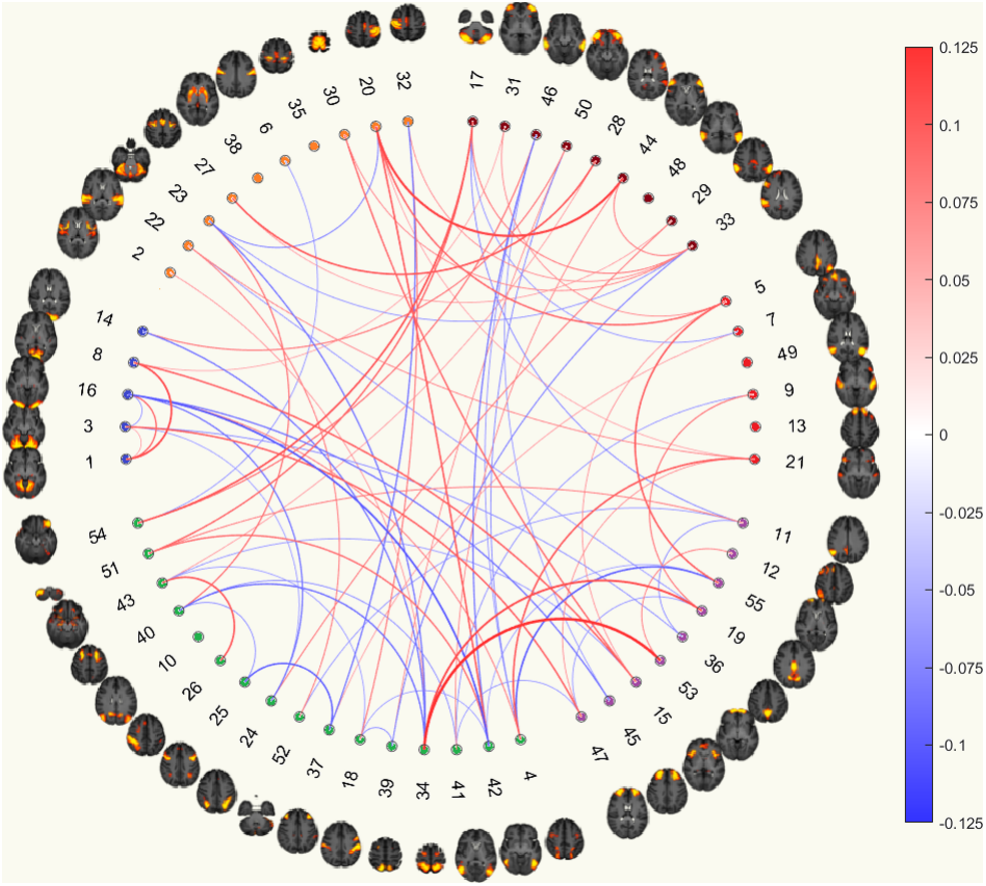
(d) Currently depressed with CTQ threshold based on partial correlation functional connectomes.

**Fig. 8.** The circular plots showing important features for predicting currently depressed phenotype with and without the CTQ threshold based on functional connectomes. The ordering of the 55 good ICA nodes is the same as that from the UKB functional network interactive visualisation, where the coloured clusters approximately maps to SMN (orange), VN (blue), EC_AN (green), CON (purple), DMN (red), and eDMN (brown). Details of method for identifying important features are presented in the Methods section

#### 3.8.2 Partial correlation matrices

For partial correlation matrices, there were significantly more edges selected as important features from the connections within VN for both the NoCTQmodel and the CTQmodel as mentioned in the above section. In addition, the connections were also more frequently selected from between SMN and eDMN, between VN and EC_AN, between VN and CON, between EC_AN and eDMN, between CON and DMN, and within eDMN for both models with matching signs.

On the other hand, we saw that the connections between the CON and eDMN were significantly more frequently selected as an important feature for the NoCTQmodel while it was less frequently selected in the CTQmodel. Moreoever, the connections were more frequently selected from within SMN and between DMN and eDMN for the NoCTQmodel but not for the CTQmodel, see Figure 8c – 8d.

### 3.9 Overall findings in functional connectomes

The important features indicated by the models were largely different across different MDD phenotypes. The connections with SMN and VN, whether it was within subnetwork connections or with other subnetworks, were selected more frequently as important features for predicting MDD phenotypes, while past studies mainly found aberrant functional connectivity within the DMN in depressed patients (Kaiser et al., 2015; Mulders et al., 2015). We are aware of the fact that these results are presented in the context of performing ML classifications, and the important features being selected here only implied that they were the edges consistently having top rankings in terms of coefficient magnitudes across folds. Therefore, the results presented in the current study do not necessarily contradict the previously reported abnormal functional connectivity within DMN in MDD patients—shared covariance for functional connectivity in these networks may well have been more consistently and strongly represented (independently of all other features) in other edges as selected by the ML classifiers across the nested cross-validation folds. As far as the effect of CTQ thresholding is concerned, in addition to the low Jaccard index seen in Table 3a and Table 3b, some of the overlapping edges had coefficients with opposing associations. The models with CTQ thresholding usually had more important edges selected, which indicated that the magnitudes and direction of the models’ coefficients were more consistent for classifying MDD with a CTQ threshold, giving partial evidence to the claim that MDD with early adversity features forms a more homogeneous subgroup.

## 4 Discussion

In the current study, we applied logistic ridge regression to classify depression phenotypes based on functional and structural connectomes, where we ensured the 1-to-1 case-control matching and correction for confounders throughout the classification analyses for all the different MDD phenotypes. In general, the MDD classification test accuracies based on functional connectomes were higher than those based on structural connectomes, though accuracy was not >62% for any model. The classification test accuracies for Currently Depressed phenotype were the highest among all the MDD phenotypes. We also found that adding self-reported childhood adversity information to the depression case criterion benefited model accuracy for some of the models, namely Currently Depressed phenotype based on both functional and structural connectomes except for MD and ICVF, Ever Severely Depressed phenotype based on functional connectomes but not for structural connectomes, and Probable Moderate/Severe Depression phenotype based on MD, FA, ICVF, and ISOVF.

We found that ML models based on SC connectomes exhibited the lowest accuracies among all the structural network weightings. Previous research usually found SC (or edge density, a variant of SC) had stronger associations with other clinical and behavioural phenotypes (Oestreich et al., 2019). Buchanan et al. (2020) have commented that the confounding effect of age, sex, and head size on SC weightings could be considerably larger than on other types of network weightings, which may have contributed to the superior predictive performances of SC when predicting psychiatric disorders shown in previous studies (Payabvash et al., 2019; Raji et al., 2020). In the current study, we chose healthy controls with age, sex, and ICV matched with (or closest to) the cases in order to minimise the confounding effect. This could be the reason for having results inconsistent with the previous literature. In addition, a similar phenomenon was seen in our previous paper where model classification performances on general cognitive function and general psychopathology were comparable for all the structural connectivity modalities when age and sex were added to the models (Yeung et al., 2022).

In terms of MDD phenotyping, we found that the more severe phenotype usually had higher classification accuracies. It is possible that there is less heterogeneity in the more severe MDD group and therefore easier to classify. It is worth highlighting that we saw moderate-to-strong negative correlations between test accuracies and sample size, which is consistent with the results in Stolicyn et al. (2020).

When considering the important network features, we found that less than 10% of the total number of edges were considered as important features for predicting any type of depression phenotype based on any connectome modality in this study. We also found that the set of important features were different between models with and without CTQ threshold. This may have partly demonstrated the neurobiological differences between MDD patients with and without self-reported exposure to childhood trauma reported in previous studies (Fadel et al., 2021; Luo, Chen, Li, Wu, Lin, Yao, Yu, Peng, et al., 2022), and we showed this in a much larger dataset than in previous studies. However, it is also possible that by adding an addition CTQ thresholding constraint on the MDD cases, we are essentially defining a more severe type of MDD based on the given MDD phenotype (Huh et al., 2017). This may explain why there is higher similarity, higher Jaccard index, between the feature sets identified by the CTQmodels and the NoCTQmodel for the more severe MDD phenotypes. Utilising more detailed and comprehensive CTQ phenotyping as well as other main risk factors may be a potential future research direction in finding biologically driven MDD subtypes, as well as subtyping for anxiety disorder and other trauma-related disorders.

For resting-state functional connectivity, in contrast to most previous studies where they either found significant hyperconnectivity or hypoconnectivity within DMN in depressed patients (Hamani et al., 2011; Kaiser et al., 2015; Sheline et al., 2010; Yan et al., 2019), we found that the DMN was not often selected as the hub of important connections for classifying MDD, and sometimes even less than random chance. The connections within the DMN are not selected significantly more often than random chance in any of the MDD phenotypes based on either pCorr or Corr, except for the Ever Depressed phenotype with CTQ threshold of 0.4 based on pCorr where connections within the DMN are negatively predictive for MDD. On the other hand, we found that the connections from as well as within SMN and VN, although they were of small effects (i.e., small magnitudes in model coefficients), were more frequently selected as important features for predicting MDD than other subnetworks. For the models which indicated SMN and VN as important hubs for MDD classification, the connectivity within SMN and VN was mostly positive predictive for MDD. The fact that connections from SMN and VN were more frequently selected by the models as important features for classifying MDD across different MDD phenotypes possibly suggests that differences in networks involved in processing of sensory information may be a more stable neuroimaging marker for the purposes of MDD prediction out-of-sample (Javaheripour et al., 2021). We are aware that the results only implied variations in these areas best differentiate MDD cases from controls in the context of classification modelling. Replication in other community samples is needed to assess the robustness of the results from the current study.

There are a few limitations to this study. First, different atlas for parcellation of the nodes for the structural and functional connectomes at the preprocessing step, where group Independent Component Analysis (group-ICA) was used for functional connectomes and the Desikan-Killiany atlas was used for the structural connectomes. This partly limits direct neuroanatomical comparisons of selected features for structural and functional connectivity and interpretations on the added value of combining structural and functional connectivity in MDD classifications. Second, representing functional connectivity in the form of one adjacency matrix per person may not be the most optimal way. There are studies with smaller data sets which use functional connectomes in time series form and applied the hybrid model, combining Graph Convolutional Neural Network (GCNN) and Long-Short Term Memory (LSTM) Network for predicting clinical phenotypes. They reported that the hybrid models performed better at classifying autistic patients compared to other ML models (Masood & Kashef, 2022; Wang et al., 2021). Similarly, structural network measures are known to be sensitive to the network construction methodology (Qi et al., 2015). Therefore, different structural findings might be obtained with connectome methods different to those applied here. Fourth, the CTQ items from the UKB have an abbreviated scale and are potentially confounded by current mood, which may potentially introduce bias (Madden et al., 2022). In addition, different types of trauma are likely to have differential effect on MDD individuals. Certain types of childhood trauma have been suggested to be more associated with symptom severity in MDD patients, while different types of trauma have been linked to differential alteration in MDD brain functional connectivity (Fadel et al., 2021; Negele et al., 2015). A more comprehensive and detailed CTQ is needed to thoroughly investigate the effect of childhood adversity on depression. Moreover, the UKB consists of healthier, wealthier, and older individuals (Fry et al., 2017) and only a small portion of the MDD cases meet the criteria for current depression. Although it was found that the currently depressed phenotype achieved the best classification accuracies, the small sample size could have been the reason for the high accuracy, as shown in the Results. Similar results were also shown in a previous study (Stolicyn et al., 2020). These results provide the basis for further replication and refinement in other community samples and in clinical cohorts.

## 5 Conclusion

In conclusion, this study reports a comprehensive data-driven MDD classification analysis, classifying six MHQ-derived MDD phenotypes based on a wide range of functional and structural brain connectivity measures, on a large community sample (UKB). The results indicated a positive relationship between depression severity and classification accuracy. Our findings also suggested that SMN and VN may be more robust biomarkers of MDD than other resting-state networks. The model for Currently Depressed phenotype based on pCorr achieved the highest accuracy of 60.2%, and the accuracy for classifying this phenotype with a CTQ threshold was 4.6% higher. The set of important features selected for models with and without CTQ threshold were largely different. Connections from SMN and VN were more frequently selected for classifying MDD across different MDD phenotypes rather than DMN and EC_AN. The robust functional connectivity features for MDD classification identified in the current study were in line with the recent imaging studies in MDD. This provided the basis to verify and build on the idea of having sensory-related subnetworks as one of the possible robust biomarkers for MDD.

## Supplementary Material

Supplementary Material

## Data Availability

The UK Biobank’s Access Procedures stipulate that participant data can only be made available to approved researchers. Therefore, the data used in this study cannot be made available for public access. The R code for the Depression Medicated, Ever Depressed, Ever Severely Depressed, Currently Depressed, and Recurrent Depression phenotypes is available in Coleman and Davis (2019).
