# Supplementary material for: Classification accuracy of structural and functional connectomes across different depressive phenotypes
Source: Imaging Neurosci (Camb). 2024 Jan 17;2:imag_2_00064. doi: 10.1162/imag_a_00064 (PMC12224484; doi:10.1162/imag_a_00064)
Supplement: Supplementary Material [file imag_a_00064-supp.pdf]

Classification accuracy of structural and functional connectomes across different depressive phenotypes
Supplementary Materials

Hon Wah Yeung[1], Aleks Stolicyn[1], Xueyi Shen[1], Mark J. Adams[1], Liana Romaniuk[1], Gladi Thng[1], Colin R. Buchanan[2,8,9], Elliot M. Tucker-Drob[3,4], Mark E. Bastin[5,8,9], Andrew M. McIntosh[1,6], Simon R. Cox[2,8,9,*], Keith Smith[7,*], and Heather C. Whalley[1,*]

[1]Department of Psychiatry, University of Edinburgh, Edinburgh, United Kingdom
[2]Department of Psychology, University of Edinburgh, Edinburgh, United Kingdom
[3]Department of Psychology, University of Texas, Austin, TX, USA
[4]Population Research Center and Center on Aging and Population Sciences, University of Texas at Austin, TX, USA
[5]Centre for Clinical Brain Science, University of Edinburgh, Edinburgh, United Kingdom
[6]Centre for Genomic and Experimental Medicine, Institute of Genetics and Molecular Medicine, University of Edinburgh, Edinburgh, UK
[7]Department of Computer and Information Sciences, University of Strathclyde, Glasgow, United Kingdom
[8]Lothian Birth Cohorts, University of Edinburgh, Edinburgh, United Kingdom
[9]Scottish Imaging Network, A Platform for Scientific Excellence Collaboration (SINAPSE), Edinburgh, United Kingdom
[*]These authors share joint senior authorship

December 18, 2023

S1 Materials and Methods

S1.1 Depression Definitions

The first five definitions (Depression Medicated, Ever Depressed, Ever Severely Depressed, Currently Depressed, Recurrent Depression) were presented in Davis et al. (2020) in details, and the R code is available on (Coleman and Davis, 2019). The detail description of the last MDD phenotype, probable moderate/severe depression, was presented in Smith et al. (2013). For clarification, the brief description and the code for the six MDD phenotypes used in this study were shown below.

Table S1: Description and code for the six MDD phenotypes used in this study

MDD phenotypes	Description	Field and code
Depression Medicated	Self-reported to have taken at least one of the medications listed on the antidepressants list, Field ID: 20003, see Coleman and Davis (2019)	Please refer to the R code for the antidepressant codes
Ever Depressed	At least one core symptom of depression, most or all of the day on most or all days for a two week period, with at least five depressive symptoms that represent a change from usual occurring over the same time-scale, with some or a lot of	Persistent sadness (20446) = Yes OR Loss of interest (20441) = Yes<br>AND<br>How much of day (20436) = Most of day or All day long<br>AND<br>Did you feel this way (20439) = Almost every day or Every day

Table S1: Description and code for the six MDD phenotypes used in this study

**MDD phenotypes**	**Description**	**Field and code**
	impairment.	AND<br>Impairment (20440) = Somewhat or A lot<br>AND<br>Total number of symptoms endorsed (core and others) ≥ 5<br>Persistent sadness (core) 20446; Loss of interest (core) 20441; Tired or low energy 20449; Gain or loss of weight 20536 = Gain, Loss or Gain and loss; Sleep change 20532; Trouble concentrating 20435; Feeling worthless 20450; Thinking about death 20437
Ever Severely Depressed		{Ever Depressed}<br>AND<br>CIDI Severity Score = 8
Currently Depressed	PHQ+ve and CIDI+ve Reports symptoms in the last two weeks that have bothered them. Current depression is indicated by five or more items marked to bother at or above a certain intensity: "more than half of days" for first eight items, "some days" for last item.	{Ever Depressed}<br>AND<br>Total symptoms endorsed as occurring more than half days (or some or more days for last item) ≥ 5<br>Little interest or pleasure in doing things 20514, Feeling down, depressed, or hopeless 20510, Trouble sleeping 20517, Feeling tired 20519, Poor appetite or overeating 20511, Feeling bad about yourself 20507, Trouble concentrating 20508, Moving or speaking slowly or fidgety or restless 20518, Thoughts that you would be better off dead 20513
Recurrent Depression		{Ever Depressed}<br>AND<br>Number of episodes (20442) $>$ 1 or -999 (too many to count)<br>AND<br>NOT case bipolar type I

Table S1: Description and code for the six MDD phenotypes used in this study

MDD phenotypes	Description	Field and code
		Excluded if number of episodes missing or bipolar state missing
Probable Moderate/Severe Depression	Either satisfying the criteria for Probable recurrent major depression (moderate) or Probable recurrent major depression (severe) described in Smith et al. (2013).	Probable recurrent major depression (moderate):<br>EITHER:<br>4598 Ever depressed/down for a whole week, plus<br>4609 At least two weeks duration, plus<br>4620 At least two episodes, plus<br>2090 Ever seen a GP (but not a psychiatrist) for nerves, anxiety, depression<br>OR:<br>4631 Ever anhedonic (unenthusiasm / uninterest) for a whole week, plus<br>5375 At least two weeks, plus<br>5386 At least two episodes, plus<br>2090 Ever seen a GP (but not a psychiatrist) for nerves, anxiety, depression<br><br>Probable recurrent major depression (severe):<br>EITHER:<br>4598 Ever depressed/down for a whole week, plus<br>4609 At least two weeks duration, plus<br>4620 At least two episodes, plus<br>2100 Ever seen a psychiatrist for nerves, anxiety, depression<br>OR:<br>4631 Ever anhedonic (unenthusiasm / uninterest) for a whole week, plus<br>5375 At least two weeks, plus<br>5386 At least two episodes, plus

Table S1: Description and code for the six MDD phenotypes used in this study

MDD phenotypes	Description	Field and code
		2100 Ever seen a psychiatrist for nerves, anxiety, depression

S1.2 Childhood Trauma Questionnaire

The UK Biobank Childhood Trauma Questionnaire items are listed in the following:

1. Felt loved as a child (inverse scored)
2. Someone to take me to the doctor as a child (inverse scored)
3. Sexually molested as a child
4. Physically abused by family as a child
5. Felt hated by family member as a child

which they correspond to emotional neglect, physical neglect, sexual abuse, physical abuse and emotional abuse respectively. We converted the responses to a scoring system of 0-to-4 to represent the severity (ranging from 'never true' to 'very often true') of trauma for each of the items. As for two of the positive items, it was score in an inverse way to capture trauma.

S1.3 Jaccard Index

Jaccard index is used to measure the similarity between two set of elements, and the similarity between set A and B is given by:

$$Jaccard(A, B) = \frac{|A \cap B|}{|A \cup B|} \tag{S1.1}$$

which is the size of intersection divided by the size of union.

S1.4 Predictive coefficients normalisation by subnetwork size

A straight forward aggregation of predictive coefficient of the robust edge features to subnetwork level can be computed using the following:

$$S = C^T E C \tag{S1.2}$$

where E is a n-by-n predictive coefficients matrix for the robust edges (n is the number of nodes), S is a c-by-c matrix (c is the number of subnetworks), S_{ij} represents the aggregated predictive coefficient for the connectivity between subnetwork i and j, C is n-by-c community indicator matrix, $C_{ij} = \delta(c_i, c_j)$ with c_i representing the community of node i.

However, this is a biased measurement due to the size differences among the subnetworks. To account for this, we replace C by C', where

$$C'_{ij} = \frac{C_{ij}}{\sqrt{\sum_{k=1}^{n} C_{kj}}} \tag{S1.3}$$

S2 MDD phenotypes Classification Results

Table S2: Classification performance for different depression phenotypes (with different CTQ thresholds) based on different connectome modalities. Drug = Depression Medicated, Ever = Ever Depressed, Ever Severe = Ever Severely Depressed, Current = Currently Depressed, Recurrent = Recurrent Depression without Bipolar Disorder, MDD Narrow = Probable Moderate/Severe Depression

MDD Phenotype	Drug	Ever	Ever Severe	Current	Recurrent	MDD narrow
Without CTQ Threshold	54.59 (3.88)	53.07 (1.02)	53.31 (3.44)	54.40 (6.88)	53.60 (2.07)	54.06 (2.70)
CTQ Threshold at 0.2	53.94 (3.51)	52.53 (1.83)	52.99 (3.36)	58.30 (4.69)	51.83 (2.23)	53.86 (4.25)
CTQ Threshold at 0.4	53.94 (4.04)	53.94 (1.17)	54.57 (4.56)	58.61 (6.84)	52.58 (1.64)	53.82 (2.61)
CTQ Threshold at 0.6	53.85 (4.44)	52.56 (2.43)	54.79 (5.86)	57.13 (5.59)	53.32 (3.55)	54.25 (3.45)

(a) Classification accuracies of the test set (mean percentage with standard deviation in brackets) for different depression phenotypes (with different CTQ thresholds) based on correlations functional connectome

MDD Phenotype	Drug	Ever	Ever Severe	Current	Recurrent	MDD narrow
Without CTQ Threshold	57.71 (3.42)	53.99 (1.38)	53.74 (3.74)	60.24 (5.15)	55.18 (1.72)	52.60 (3.11)
CTQ Threshold at 0.2	57.83 (2.65)	52.98 (1.68)	54.63 (3.51)	61.72 (4.92)	51.61 (2.24)	52.25 (4.27)
CTQ Threshold at 0.4	56.51 (3.42)	53.30 (2.14)	55.30 (3.74)	64.76 (4.36)	52.05 (2.36)	51.98 (3.31)
CTQ Threshold at 0.6	56.60 (3.98)	52.32 (2.12)	55.25 (4.36)	60.34 (6.93)	52.35 (2.56)	52.41 (4.18)

(b) Classification accuracies of the test set (mean percentage with standard deviation in brackets) for different depression phenotypes (with different CTQ thresholds) based on partial correlations functional connectome

MDD Phenotype	Drug	Ever	Ever Severe	Current	Recurrent	MDD narrow
Without CTQ Threshold	54.79 (3.49)	51.24 (1.77)	53.37 (6.42)	57.37 (6.26)	52.80 (2.69)	53.72 (4.78)
CTQ Threshold at 0.2	53.55 (4.40)	52.04 (2.13)	51.04 (4.92)	54.74 (9.83)	52.76 (3.40)	52.31 (4.16)
CTQ Threshold at 0.4	52.47 (5.60)	52.14 (2.23)	50.24 (6.42)	56.06 (7.98)	51.87 (4.38)	56.07 (5.55)
CTQ Threshold at 0.6	53.80 (6.52)	52.96 (2.62)	52.03 (5.52)	54.55 (7.37)	50.15 (4.36)	55.43 (6.70)

(c) Classification accuracies of the test set (mean percentage with standard deviation in brackets) for different depression phenotypes (with different CTQ thresholds) based on mean diffusivity structural connectome

MDD Phenotype	Drug	Ever	Ever Severe	Current	Recurrent	MDD narrow
Without CTQ Threshold	55.00 (4.22)	51.00 (1.94)	54.96 (4.47)	56.82 (9.23)	53.65 (3.98)	53.63 (4.03)
CTQ Threshold at 0.2	55.13 (5.50)	52.49 (2.49)	51.70 (5.04)	55.38 (10.08)	52.72 (3.75)	53.97 (4.47)
CTQ Threshold at 0.4	51.50 (7.54)	52.55 (2.87)	50.65 (6.64)	59.24 (9.32)	53.44 (3.25)	55.52 (7.62)
CTQ Threshold at 0.6	53.82 (7.39)	52.70 (2.87)	54.10 (9.18)	60.48 (10.44)	53.35 (4.01)	56.08 (7.96)

(d) Classification accuracies of the test set (mean percentage with standard deviation in brackets) for different depression phenotypes (with different CTQ thresholds) based on fractional anisotropy structural connectome

Table S2: Classification performance for different depression phenotypes (with different CTQ thresholds) based on different connectome modalities. Drug = Depression Medicated, Ever = Ever Depressed, Ever Severe = Ever Severely Depressed, Current = Currently Depressed, Recurrent = Recurrent Depression without Bipolar Disorder, MDD Narrow = Probable Moderate/Severe Depression (Cont.)

MDD Phenotype	Drug	Ever	Ever Severe	Current	Recurrent	MDD narrow
Without CTQ Threshold	52.44 (4.88)	51.00 (2.44)	53.46 (4.44)	53.43 (8.63)	51.14 (2.60)	49.88 (3.11)
CTQ Threshold at 0.2	49.76 (3.75)	50.86 (1.97)	51.90 (4.64)	52.82 (6.30)	51.11 (3.73)	49.37 (4.62)
CTQ Threshold at 0.4	48.68 (8.02)	50.39 (2.81)	52.77 (5.63)	55.69 (7.20)	50.12 (3.78)	47.82 (4.68)
CTQ Threshold at 0.6	50.79 (5.23)	50.04 (3.06)	53.73 (8.15)	54.88 (8.34)	50.40 (3.51)	45.92 (5.49)

(e) Classification accuracies of the test set (mean percentage with standard deviation in brackets) for different depression phenotypes (with different CTQ thresholds) based on streamline count structural connectome

MDD Phenotype	Drug	Ever	Ever Severe	Current	Recurrent	MDD narrow
Without CTQ Threshold	53.37 (5.14)	51.39 (1.94)	54.11 (4.43)	53.39 (7.24)	51.93 (3.07)	51.05 (4.09)
CTQ Threshold at 0.2	54.97 (4.44)	50.90 (1.90)	50.20 (4.22)	53.59 (6.85)	51.27 (3.58)	51.02 (4.84)
CTQ Threshold at 0.4	52.74 (7.04)	51.59 (2.90)	50.89 (5.06)	57.39 (7.20)	51.87 (4.07)	51.07 (5.60)
CTQ Threshold at 0.6	52.39 (6.10)	51.28 (3.12)	54.28 (4.63)	54.24 (6.89)	50.95 (4.96)	49.96 (6.33)

(f) Classification accuracies of the test set (mean percentage with standard deviation in brackets) for different depression phenotypes (with different CTQ thresholds) based on orientation dispersion structural connectome

MDD Phenotype	Drug	Ever	Ever Severe	Current	Recurrent	MDD narrow
Without CTQ Threshold	52.62 (3.89)	51.64 (1.88)	50.61 (4.11)	57.58 (4.97)	52.03 (2.90)	51.20 (5.89)
CTQ Threshold at 0.2	52.57 (5.41)	53.55 (2.18)	51.48 (5.73)	57.31 (7.58)	53.56 (4.21)	52.05 (4.72)
CTQ Threshold at 0.4	52.45 (7.13)	53.58 (2.17)	51.79 (5.72)	60.87 (10.86)	52.39 (3.58)	53.25 (4.73)
CTQ Threshold at 0.6	49.04 (6.51)	53.64 (3.05)	50.60 (4.88)	60.00 (9.54)	51.94 (3.84)	53.03 (7.58)

(g) Classification accuracies of the test set (mean percentage with standard deviation in brackets) for different depression phenotypes (with different CTQ thresholds) based on isotropic volume fraction structural connectome

MDD Phenotype	Drug	Ever	Ever Severe	Current	Recurrent	MDD narrow
Without CTQ Threshold	56.06 (4.46)	52.67 (1.81)	55.85 (5.36)	60.00 (10.78)	53.82 (2.98)	54.06 (4.10)
CTQ Threshold at 0.2	52.72 (6.42)	52.65 (2.10)	54.77 (5.21)	60.13 (8.72)	52.30 (3.16)	54.63 (4.20)
CTQ Threshold at 0.4	51.48 (7.19)	54.12 (2.48)	53.70 (7.01)	59.70 (10.12)	54.07 (3.54)	57.90 (5.94)
CTQ Threshold at 0.6	55.49 (6.57)	53.54 (3.06)	54.91 (7.34)	58.40 (7.89)	53.81 (5.11)	56.37 (7.93)

(h) Classification accuracies of the test set (mean percentage with standard deviation in brackets) for different depression phenotypes (with different CTQ thresholds) based on intra-cellular volume fraction structural connectome

S2.1 MDD Classification results for sex specific models based on functional conectomes

Table S3: Classification performance of sex specific model for different depression phenotypes (with different CTQ thresholds) based on functional connectomes. Drug = Depression Medicated, Ever = Ever Depressed, Ever Severe = Ever Severely Depressed, Current = Currently Depressed, Recurrent = Recurrent Depression without Bipolar Disorder, MDD Narrow = Probable Moderate/Severe Depression

Female MDD Classification Models

MDD Phenotype	Medicated	Ever	Ever Severe	Current	Recurrent	MDD narrow
Without CTQ Threshold	54.71 (3.23)	53.26 (1.44)	53.37 (3.49)	56.34 (7.16)	53.73 (2.08)	55.04 (2.33)
CTQ Threshold at 0.2	54.12 (5.09)	53.11 (2.64)	54.54 (5.91)	57.59 (5.90)	53.19 (2.49)	55.71 (3.28)
CTQ Threshold at 0.4	52.62 (5.98)	53.84 (2.04)	53.24 (5.13)	55.20 (6.97)	53.49 (2.47)	53.68 (3.98)
CTQ Threshold at 0.6	50.83 (5.85)	53.47 (3.04)	56.77 (6.10)	55.32 (10.68)	54.36 (3.20)	53.98 (3.77)

(a) Classification accuracies of the test set (mean percentage with standard deviation in brackets) for different depression phenotypes (with different CTQ thresholds) for female MDD models based on correlation functional connectomes.

MDD Phenotype	Medicated	Ever	Ever Severe	Current	Recurrent	MDD narrow
Without CTQ Threshold	58.57 (5.13)	53.56 (1.42)	55.24 (3.25)	59.37 (7.15)	54.37 (2.02)	53.03 (2.63)
CTQ Threshold at 0.2	58.45 (4.45)	52.77 (2.12)	54.03 (5.12)	60.63 (7.85)	52.55 (2.44)	53.31 (3.54)
CTQ Threshold at 0.4	55.50 (4.82)	52.75 (1.48)	57.67 (3.94)	63.42 (12.01)	52.41 (2.81)	50.72 (4.28)
CTQ Threshold at 0.6	53.89 (6.04)	52.99 (1.89)	56.87 (5.60)	65.93 (10.27)	51.39 (3.40)	53.76 (5.03)

(b) Classification accuracies of the test set (mean percentage with standard deviation in brackets) for different depression phenotypes (with different CTQ thresholds) for female MDD models based on partial correlation functional connectomes.

Male MDD Classification Models

MDD Phenotype	Medicated	Ever	Ever Severe	Current	Recurrent	MDD narrow
Without CTQ Threshold	51.85 (5.07)	50.25 (2.52)	53.91 (5.78)	54.58 (10.29)	49.76 (2.90)	49.37 (5.46)
CTQ Threshold at 0.2	50.79 (8.63)	49.20 (2.20)	52.12 (6.54)	55.18 (8.92)	47.96 (3.40)	51.15 (3.68)
CTQ Threshold at 0.4	53.66 (6.93)	48.05 (3.14)	48.89 (9.26)	57.22 (8.89)	46.38 (4.74)	49.44 (6.24)
CTQ Threshold at 0.6	57.79 (8.10)	47.98 (4.08)	49.19 (9.97)	55.83 (7.93)	46.33 (5.24)	49.46 (6.66)

(c) Classification accuracies of the test set (mean percentage with standard deviation in brackets) for different depression phenotypes (with different CTQ thresholds) for male MDD models based on correlation functional connectomes.

MDD Phenotype	Medicated	Ever	Ever Severe	Current	Recurrent	MDD narrow
Without CTQ Threshold	54.00 (5.40)	52.08 (2.39)	52.80 (5.51)	56.67 (8.86)	52.05 (3.43)	52.49 (5.44)
CTQ Threshold at 0.2	50.69 (6.96)	50.62 (3.12)	48.53 (6.55)	58.52 (8.32)	48.20 (4.27)	52.32 (5.31)
CTQ Threshold at 0.4	53.22 (10.68)	48.82 (2.71)	47.14 (8.08)	57.96 (8.72)	44.97 (5.94)	51.16 (8.09)
CTQ Threshold at 0.6	57.24 (11.62)	49.89 (4.70)	45.19 (9.12)	53.07 (12.04)	44.92 (5.95)	53.01 (9.87)

(d) Classification accuracies of the test set (mean percentage with standard deviation in brackets) for different depression phenotypes (with different CTQ thresholds) for male MDD models based on partial correlation functional connectomes.

S3 Hypothesis testing of feature occurrence in subnetworks and normalised predictive coefficients for different MDD phenotypes

Table S4: Normalised predictive coefficient between or within subnetworks for the six MDD classification without CTQ threshold based on Corr functional connectomes. The uncorrected p-values from hypergeometric test were in the brackets. Cells with p-values < 0.05 were highlighted in red or green where red cell = fewer edges, green cell = more edges. None of the p-values survived the FDR corrected threshold.

Functional Subnetwork	SMN	VN	EC_AN	CON	DMN	eDMN
SMN	-0.0127 (0.0926)					
VN	0.0069 (0.6562)	0.0204 (0.2182)				
EC_AN	-0.0166 (0.3453)	0.0030 (0.3060)	0.0076 (0.5674)			
CON	0.0025 (0.3743)	-0.0129 (0.2984)	-0.0052 (0.5321)	0 (0.4089)		
DMN	0.0143 (0.4318)	0 (0.4754)	0 (0.0875)	0.0013 (0.3796)	0.0113 (0.3092)	
eDMN	0.0003 (0.6257)	0.0095 (0.7016)	-0.0015 (0.4679)	0 (0.1295)	0 (0.2593)	0 (0.4089)

(a) DEPRESSION MEDICATED

Functional Subnetwork	SMN	VN	EC_AN	CON	DMN	eDMN
SMN	0.0069 (0.2656)					
VN	0 (0.7092)	0.0102 (0.0655)				
EC_AN	0.0023 (0.7060)	0.0037 (0.4262)	0 (0.4294)			
CON	-0.0028 (0.4659)	0.0043 (0.2656)	0 (0.3594)	-0.0079 (0.2182)		
DMN	0 (0.6612)	0 (0.8149)	-0.0033 (0.4885)	0 (0.6897)	0 (0.9032)	
eDMN	-0.0069 (0.1191)	0 (0.7344)	0 (0.3594)	0 (0.5697)	0 (0.6897)	0 (0.7818)

(b) EVER DEPRESSED

Functional Subnetwork	SMN	VN	EC_AN	CON	DMN	eDMN
SMN	-0.0198 (0.6304)					
VN	0.0141 (0.7067)	0.0725 (0.0182)				
EC_AN	0.0075 (0.1239)	-0.0018 (0.5226)	0.0204 (0.0389)			
CON	-0.0079 (0.4117)	-0.0205 (0.0699)	-0.0131 (0.3858)	0 (0.4522)		
DMN	0 (0.2634)	0 (0.5168)	-0.0257 (0.6576)	0.0105 (0.6742)	0.0543 (0.0397)	
eDMN	-0.0195 (0.5883)	0 (0.3696)	0.0151 (0.3858)	0.0190 (0.4705)	0.0161 (0.1074)	0 (0.4522)

(c) EVER SEVERELY DEPRESSED

Functional Subnetwork	SMN	VN	EC_AN	CON	DMN	eDMN
SMN	0.0117 (0.4489)					
VN	-0.0007 (0.6713)	0.0812 (0.0586)				
EC_AN	-0.0113 (0.5267)	-0.0174 (0.3610)	-0.0052 (0.5420)			
CON	-0.0019 (0.2817)	-0.0217 (0.5511)	-0.0030 (0.3619)	-0.0132 (0.1752)		
DMN	-0.0007 (0.5590)	0 (0.2866)	-0.0011 (0.2408)	0.0249 (0.3741)	0 (0.5371)	
eDMN	0.0162 (0.4993)	-0.0019 (0.5511)	0.0290 (0.2194)	-0.0080 (0.5837)	-0.0138 (0.1705)	0 (0.2225)

(d) CURRENTLY DEPRESSED

Functional Subnetwork	SMN	VN	EC_AN	CON	DMN	eDMN
SMN	-0.0282 (0.3904)					
VN	0 (0.5765)	0 (0.8970)				
EC_AN	-0.0213 (0.5279)	0.0121 (0.5896)	0.0175 (0.6246)			
CON	0 (0.3658)	0.0207 (0.3904)	-0.0065 (0.5306)	0 (0.6739)		
DMN	0 (0.5151)	0 (0.7202)	-0.0104 (0.0794)	0.0167 (0.4488)	0 (0.8494)	
eDMN	-0.0304 (0.2524)	0 (0.6096)	0.0144 (0.4694)	0 (0.4057)	0 (0.5512)	0.0260 (0.3261)

(e) RECURRENT DEPRESSION

Functional Subnetwork	SMN	VN	EC_AN	CON	DMN	eDMN
SMN	0.0045 (0.2984)					
VN	0.0060 (0.6562)	0 (0.7818)				
EC_AN	-0.0204 (0.3453)	-0.0027 (0.3060)	-0.0041 (0.4326)			
CON	-0.0013 (0.1698)	-0.0148 (0.2984)	-0.0110 (0.4679)	0.0194 (0.0540)		
DMN	0 (0.2225)	0 (0.4754)	0 (0.0875)	0.0053 (0.6204)	0 (0.6908)	
eDMN	0.0092 (0.1698)	0 (0.3259)	-0.0074 (0.3051)	0.0074 (0.4053)	0.0052 (0.6204)	-0.0107 (0.5911)

(f) PROBABLE MODERATE/SEVERE DEPRESSION

Table S5: Normalised predictive coefficient between or within subnetworks for the six MDD classification with CTQ threshold at 0.4 based on Corr functional connectomes. The uncorrected p-values from hypergeometric test were in the brackets. Cells with p-values < 0.05 were highlighted in red or green where red cell = fewer edges, green cell = more edges, bold text with asterisk = remain significant after FDR correction.

Functional Subnetwork	SMN	VN	EC_AN	CON	DMN	eDMN
SMN	-0.0292 (0.1050)					
VN	-0.0061 (0.1331)	0 (0.7710)				
EC_AN	-0.0007 (0.6096)	-0.0051 (0.3822)	0.0064 (0.3691)			
CON	-0.0053 (0.3170)	0 (0.3059)	-0.0067 (0.4883)	0 (0.3889)		
DMN	0.0141 (0.4598)	0 (0.4559)	0.0084 (0.2822)	-0.0058 (0.1572)	0 (0.6766)	
eDMN	0.0081 (0.4081)	0.0228 (0.0256)	-0.0011 (0.3054)	-0.0065 (0.3753)	0 (0.2403)	0 (0.3889)

(a) DEPRESSION MEDICATED

Functional Subnetwork	SMN	VN	EC_AN	CON	DMN	eDMN
SMN	-0.0029 (0.0548)					
VN	0.0007 (0.2552)	0.0051 (0.1795)				
EC_AN	-0.0012 (0.1952)	0.0018 (0.5304)	0.0046 (0.5801)			
CON	-0.0013 (0.4662)	0.0030 (0.5939)	-0.0017 (0.2107)	0 (0.4874)		
DMN	-0.0001 (0.3289)	0 (0.5501)	-0.0014 (0.4302)	0 (0.3380)	0 (0.7429)	
eDMN	-0.0005 (0.0938)	0 (0.4061)	0.0011 (0.5451)	-0.0018 (0.5238)	-0.0023 (0.7153)	0 (0.4874)

(b) EVER DEPRESSED

Functional Subnetwork	SMN	VN	EC_AN	CON	DMN	eDMN
SMN	0 (0.3936)					
VN	-0.0107 (0.7321)	0 (0.8149)				
EC_AN	-0.0110 (0.3562)	0.0200 (0.4877)	0.0205 (0.5578)			
CON	-0.0016 (0.5525)	-0.0348 (0.0597)	0.0073 (0.5689)	0.0221 (0.5246)		
DMN	0 (0.2866)	0.0142 (0.4612)	-0.0135 (0.5887)	0.0117 (0.7016)	0.0185 (0.2648)	
eDMN	-0.0173 (0.5525)	-0.0160 (0.6064)	0.0006 (0.4311)	0.0116 (0.5056)	0.0192 (0.2984)	0.0124 (0.5246)

(c) EVER SEVERELY DEPRESSED

Functional Subnetwork	SMN	VN	EC_AN	CON	DMN	eDMN
SMN	-0.0066 (0.4217)					
VN	-0.0065 (0.3571)	**0.0874 (0.0005)***				
EC_AN	0.0141 (0.1718)	-0.0061 (0.0484)	-0.0038 (0.5994)			
CON	-0.0003 (0.2507)	-0.0131 (0.0386)	-0.0087 (0.4120)	0.0071 (0.5429)		
DMN	0.0051 (0.2652)	0 (0.2688)	-0.0079 (0.6142)	0.0138 (0.4044)	0.0096 (0.4797)	
eDMN	0.0145 (0.3334)	0.0098 (0.5783)	0.0120 (0.4157)	0 (0.0270)	-0.0153 (0.4044)	0 (0.2060)

(d) CURRENTLY DEPRESSED

Functional Subnetwork	SMN	VN	EC_AN	CON	DMN	eDMN
SMN	-0.0229 (0.4943)					
VN	0 (0.4681)	0 (0.8610)				
EC_AN	**-0.0189 (0.0062)***	0 (0.2931)	0.0459 (0.2601)			
CON	0.0110 (0.6107)	0.0362 (0.1415)	0 (0.1043)	0 (0.5805)		
DMN	0 (0.4009)	0 (0.6362)	-0.0316 (0.1658)	0 (0.4401)	0 (0.7986)	
eDMN	-0.0384 (0.3893)	0 (0.5057)	0.0055 (0.6389)	-0.0100 (0.6603)	0 (0.4401)	0 (0.5805)

(e) RECURRENT DEPRESSION

Functional Subnetwork	SMN	VN	EC_AN	CON	DMN	eDMN
SMN	0.0111 (0.0950)					
VN	0.0004 (0.5908)	0 (0.6706)				
EC_AN	-0.0111 (0.0067)	-0.0052 (0.3845)	0.0006 (0.3254)			
CON	0.0025 (0.1212)	-0.0037 (0.4676)	-0.0041 (0.0671)	0.0040 (0.5854)		
DMN	0.0025 (0.4189)	0 (0.2990)	0.0006 (0.2619)	0.0052 (0.6463)	0 (0.5485)	
eDMN	0.0060 (0.0567)	-0.0066 (0.2555)	0.0029 (0.3212)	0.0018 (0.6092)	0.0017 (0.3665)	-0.0028 (0.5854)

(f) PROBABLE MODERATE/SEVERE DEPRESSION

Table S6: Normalised predictive coefficient between or within subnetworks for the six MDD classification without CTQ threshold based on pCorr functional connectomes. The uncorrected p-values from hypergeometric test were in the brackets. Cells with p-values < 0.05 were highlighted in red or green where red cell = fewer edges, green cell = more edges. None of the p-values survived the FDR corrected threshold.

Functional Subnetwork	SMN	VN	EC_AN	CON	DMN	eDMN
SMN	-0.0194 (0.0203)					
VN	-0.0001 (0.2283)	0 (0.6031)				
EC_AN	-0.0122 (0.5380)	-0.0010 (0.1953)	0.0013 (0.5477)			
CON	-0.0030 (0.5422)	-0.0015 (0.3826)	0.0028 (0.0631)	0.0089 (0.5367)		
DMN	0.0039 (0.1932)	-0.0039 (0.5608)	0.0042 (0.0424)	-0.0015 (0.2723)	0 (0.4678)	
eDMN	0.0013 (0.5422)	0.0201 (0.1764)	-0.0006 (0.2710)	0.0004 (0.3670)	-0.0176 (0.1216)	0.0055 (0.4633)

(a) DEPRESSION MEDICATED

Functional Subnetwork	SMN	VN	EC_AN	CON	DMN	eDMN
SMN	0.0011 (0.0459)					
VN	0.0004 (0.4212)	0 (0.5418)				
EC_AN	-0.0004 (0.5191)	0.0038 (0.5207)	-0.0006 (0.4186)			
CON	-0.0015 (0.2039)	-0.0014 (0.5056)	-0.0012 (0.1263)	0.0034 (0.1603)		
DMN	-0.0002 (0.2964)	0 (0.1570)	-0.0019 (0.4897)	0.0023 (0.3677)	-0.0040 (0.6018)	
eDMN	0.0009 (0.2811)	0.0012 (0.5056)	0.0105 (0.1364)	-0.0017 (0.3465)	0.0034 (0.6006)	0.0043 (0.6394)

(b) EVER DEPRESSED

Functional Subnetwork	SMN	VN	EC_AN	CON	DMN	eDMN
SMN	-0.0126 (0.5684)					
VN	-0.0042 (0.4868)	0.0701 (0.0390)				
EC_AN	-0.0075 (0.2211)	-0.0088 (0.4844)	-0.0008 (0.1858)			
CON	0.0181 (0.4340)	-0.0016 (0.4316)	-0.0111 (0.2991)	0.0201 (0.6743)		
DMN	0.0224 (0.1250)	0 (0.3695)	-0.0279 (0.3767)	0.0334 (0.2522)	0.0228 (0.3906)	
eDMN	-0.0158 (0.4340)	0.0081 (0.5684)	-0.0147 (0.4950)	0.0076 (0.4928)	-0.0099 (0.4714)	0 (0.3020)

(c) EVER SEVERELY DEPRESSED

Functional Subnetwork	SMN	VN	EC_AN	CON	DMN	eDMN
SMN	-0.0034 (0.4079)					
VN	0 (0.0691)	0.0271 (0.0891)				
EC_AN	0.0130 (0.5646)	-0.0029 (0.3902)	-0.0048 (0.5825)			
CON	-0.0010 (0.5058)	-0.0040 (0.4079)	0.0090 (0.5400)	0 (0.1474)		
DMN	0.0028 (0.6310)	0 (0.2035)	0.0054 (0.1172)	0.0069 (0.2973)	0 (0.4530)	
eDMN	0.0049 (0.4942)	-0.0033 (0.3178)	0.0028 (0.3128)	-0.0137 (0.0502)	0.0006 (0.2973)	0.0004 (0.5590)

(d) CURRENTLY DEPRESSED

Functional Subnetwork	SMN	VN	EC_AN	CON	DMN	eDMN
SMN	0.0028 (0.3524)					
VN	-0.0012 (0.5695)	0.0035 (0.4995)				
EC_AN	0.0007 (0.1026)	0.0059 (0.2810)	-0.0038 (0.1705)			
CON	-0.0055 (0.6068)	-0.0003 (0.4121)	0.0028 (0.3630)	0.0004 (0.5650)		
DMN	0.0034 (0.3708)	0 (0.1236)	-0.0011 (0.2163)	-0.0014 (0.1125)	-0.0053 (0.2640)	
eDMN	0.0006 (0.3932)	0.0027 (0.3524)	0.0032 (0.1537)	0.0046 (0.2919)	0.0009 (0.1125)	0.0033 (0.5650)

(e) RECURRENT DEPRESSION

Functional Subnetwork	SMN	VN	EC_AN	CON	DMN	eDMN
SMN	0.0177 (0.4316)					
VN	-0.0070 (0.2172)	0 (0.7192)				
EC_AN	0.0018 (0.4159)	-0.0076 (0.5156)	-0.0222 (0.0862)			
CON	0.0033 (0.1989)	0 (0.2228)	-0.0115 (0.1764)	0.0089 (0.1075)		
DMN	0.0115 (0.5870)	-0.0151 (0.2526)	-0.0066 (0.1942)	-0.0041 (0.4714)	0 (0.6094)	
eDMN	0.0055 (0.1989)	0 (0.2228)	0.0156 (0.4950)	-0.0061 (0.2503)	0.0044 (0.4714)	0 (0.3020)

(f) PROBABLE MODERATE/SEVERE DEPRESSION

Table S7: Normalised predictive coefficient between or within subnetworks for the six MDD classification with CTQ threshold at 0.4 based on pCorr functional connectomes. The uncorrected p-values from hypergeometric test were in the brackets. Cells with p-values < 0.05 were highlighted in red or green where red cell = fewer edges, green cell = more edges, bold text with asterisk = remain significant after FDR correction.

Functional Subnetwork	SMN	VN	EC_AN	CON	DMN	eDMN
SMN	-0.0115 (0.3401)					
VN	-0.0067 (0.1924)	0.0199 (0.3751)				
EC_AN	0.0013 (0.1057)	-0.0055 (0.1072)	0.0474 (0.0421)			
CON	-0.0144 (0.6042)	-0.0081 (0.3791)	0.0155 (0.0888)	0.0111 (0.5022)		
DMN	-0.0024 (0.4742)	0 (0.2416)	0.0003 (0.1697)	-0.0104 (0.2802)	0 (0.4934)	
eDMN	-0.0022 (0.6042)	0.0384 (0.0511)	-0.0055 (0.5051)	0.0093 (0.2692)	-0.0258 (0.0967)	-0.0022 (0.4978)

(a) DEPRESSION MEDICATED

Functional Subnetwork	SMN	VN	EC_AN	CON	DMN	eDMN
SMN	-0.0195 (0.5676)					
VN	0.0173 (0.6067)	0 (0.8319)				
EC_AN	4.62e-05 (0.3305)	0.0168 (0.5676)	0.0280 (0.6254)			
CON	0 (0.1820)	0.0153 (0.5676)	0.0242 (0.4953)	0.0252 (0.4876)		
DMN	0.0150 (0.7015)	0 (0.5735)	0.0134 (0.4697)	0 (0.3645)	-0.0706 (0.0289)	
eDMN	0.0138 (0.2219)	0.0107 (0.5676)	0.0323 (0.4953)	-0.0110 (0.5612)	-0.0190 (0.6355)	0 (0.5124)

(b) EVER DEPRESSED

Functional Subnetwork	SMN	VN	EC_AN	CON	DMN	eDMN
SMN	-0.0076 (0.5473)					
VN	-0.0156 (0.5088)	0 (0.7092)				
EC_AN	-0.0013 (0.0797)	0.0059 (0.5123)	0.0034 (0.3791)			
CON	0.0188 (0.1815)	0.0098 (0.1908)	-0.0138 (0.2683)	-0.0095 (0.6562)		
DMN	0.0077 (0.3287)	0.0139 (0.7321)	-0.0082 (0.5930)	0.0291 (0.1046)	0 (0.5968)	
eDMN	0.0101 (0.3596)	-0.0096 (0.5473)	0.0060 (0.3551)	0.0067 (0.2300)	0.0063 (0.4489)	0.0130 (0.6562)

(c) EVER SEVERELY DEPRESSED

Functional Subnetwork	SMN	VN	EC_AN	CON	DMN	eDMN
SMN	-0.0151 (0.2969)					
VN	0 (0.0620)	**0.0741 (0.0012)***				
EC_AN	0.0137 (0.5159)	-0.0283 (0.4234)	-0.0400 (0.1813)			
CON	0.0029 (0.1285)	0.0052 (0.2143)	-0.0022 (0.5060)	-0.0121 (0.2992)		
DMN	0.0288 (0.6026)	0 (0.1908)	0.0094 (0.2333)	0.0423 (0.3226)	0 (0.4387)	
eDMN	0.0369 (0.3442)	0.0202 (0.5671)	0.0213 (0.1349)	-0.0151 (0.1805)	0.0007 (0.4431)	0.0352 (0.5807)

(d) CURRENTLY DEPRESSED

Functional Subnetwork	SMN	VN	EC_AN	CON	DMN	eDMN
SMN	0.0018 (0.2755)					
VN	0 (0.3079)	0.0141 (0.2073)				
EC_AN	0.0175 (0.0239)	0 (0.1489)	0.0132 (0.2929)			
CON	0 (0.1164)	0.0124 (0.2755)	0.0041 (0.5772)	0.0158 (0.1983)		
DMN	0.0058 (0.5969)	0 (0.4957)	-0.0082 (0.6211)	0 (0.2797)	-0.0112 (0.2947)	
eDMN	-0.0014 (0.6599)	0 (0.3471)	0.0124 (0.4228)	-0.0026 (0.4371)	0 (0.2797)	-0.0067 (0.5700)

(e) RECURRENT DEPRESSION

Functional Subnetwork	SMN	VN	EC_AN	CON	DMN	eDMN
SMN	0.0084 (0.0815)					
VN	-0.0009 (0.5612)	0 (0.6848)				
EC_AN	-0.0077 (0.3803)	0 (0.0449)	-0.0147 (0.2813)			
CON	0.0065 (0.1410)	0.0149 (0.5034)	0.0192 (0.0343)	-0.0431 (0.1456)		
DMN	0.0242 (0.3854)	0 (0.3187)	-0.0069 (0.2802)	-0.0065 (0.3960)	0 (0.5662)	
eDMN	0.0087 (0.5696)	-0.0205 (0.5034)	0.0208 (0.3690)	0 (0.0431)	0 (0.1254)	0.0184 (0.3884)

(f) PROBABLE MODERATE/SEVERE DEPRESSION

References

Coleman, J. and Davis, K. (2019). Code for use in R statistics with UK Biobank Mental Health Questionnaire data.

Davis, K. A., Coleman, J. R., Adams, M., Allen, N., Breen, G., Cullen, B., Dickens, C., Fox, E., Graham, N., Holliday, J., et al. (2020). Mental health in uk biobank–development, implementation and results from an online questionnaire completed by 157 366 participants: a reanalysis. *BJPsych open*, 6(2).

Smith, D. J., Nicholl, B. I., Cullen, B., Martin, D., Ul-Haq, Z., Evans, J., Gill, J. M., Roberts, B., Gallacher, J., Mackay, D., et al. (2013). Prevalence and characteristics of probable major depression and bipolar disorder within uk biobank: cross-sectional study of 172,751 participants. *PloS one*, 8(11):e75362.
